# Optimal Sequential Fusion Kalman Filter for Multi-Sensor Linear Systems with Noise Cross-Correlated

**DOI:** 10.3390/s25216702

**Published:** 2025-11-02

**Authors:** Weichang Huang, Chenglin Wen

**Affiliations:** 1 College of Automation, Guangdong University of Petrochemical Technology, Maoming 525000, China; huangwc@gdupt.edu.cn; 2 Guangdong Provincial Key Laboratory of Petrochemical Equipment Fault Diagnosis, Guangdong University of Petrochemical Technology, Maoming 525000, China

**Keywords:** multi-sensor systems, sequential fusion filter, centralized fusion filter, filter equivalence

## Abstract

For the state estimation problem of multi-sensor linear systems with noise cross-correlated, where process noise correlates with measurement noise and measurement noises are mutually correlated, researchers have long attempted to design a sequential fusion Kalman filter that is strictly equivalent to the centralized fusion Kalman filter. To the best of our knowledge, this problem has remained unsolved. To this end, this paper designs a truly globally optimal sequential fusion filter suitable for such systems. First, an innovative method is proposed to indirectly decorrelate process noise from all measurement noise, addressing the challenge of their direct decorrelation. Then, the measurement equations are equivalently rewritten based on the Gram–Schmidt orthogonalization principle so that mutual independence among the noises is achieved. Next, a sequential fusion Kalman filter is established based on the rewritten measurement equation. Finally, the equivalence between filters is rigorously proven theoretically. To demonstrate the effectiveness of the proposed algorithms, the problem of tracking a target moving with constant velocity is considered.

## 1. Introduction

State estimation is the process of describing and reconstructing the internal state structure of a system based on its external measurement output data. Over the past few decades, state estimation problems have been present across various fields such as industry, military, and defense, and have been widely applied in areas including parameter estimation, target tracking, and aerospace [1,2,3]. The Kalman filter is one of the most important methods for state estimation and system parameter identification. It is designed for linear systems under the assumption that the process noise and measurement noise are mutually uncorrelated. By minimizing the state estimation error covariance, the Kalman filter provides an optimal, real-time recursive estimation algorithm [4]. However, in many practical applications, the process noise and measurement noise of a system are often correlated, primarily due to the influence of common underlying factors. For instance, when an aircraft is subject to random wind disturbances during flight, the wind speed not only affects the dynamic behavior described by the system model, but also impacts the accuracy of the airspeed sensor. This shared influence of wind introduces statistical correlation between the process noise in the aircraft’s state equation and the measurement noise in the airspeed sensor’s measurement equation. The problem of fusion filtering with correlated noise has attracted considerable attention from many researchers [5,6,7].

Due to the complexity of practical application scenarios, measurement systems composed of multiple spatially distributed sensors are commonly employed to estimate the states of dynamic targets. Compared with single-sensor systems, such multisensor systems offer several advantages [8,9], including enhanced system survivability; extended spatial coverage; improved reliability and reduced information ambiguity; and enhanced detection performance, leading to increased overall system robustness. For systems in which the process noise is uncorrelated with all measurement noises, and all measurement noises are mutually uncorrelated, we refer to such systems as Noise Cross-Uncorrelated Systems (NCUSs). Under the NCUS assumption, several Kalman filtering architectures—namely, the centralized fusion Kalman filter, the distributed fusion Kalman filter, and the sequential fusion Kalman filter—are theoretically equivalent in terms of estimation performance [10,11]. The centralized fusion Kalman filtering algorithm waits for all sensor measurements to be transmitted to a central processing unit before performing data fusion. Although it yields the optimal state estimate, it requires the arrival of all sensor data prior to fusion, resulting in high computational dimensionality and a significant processing load. These factors hinder the real-time implementation of the filter. In the distributed fusion Kalman filtering algorithm [12,13,14], each sensor first generates a local estimate based on its own measurements. These local estimates are then transmitted to a fusion center, where a globally optimal distributed fusion Kalman filter is constructed. In contrast, the sequential fusion Kalman filtering algorithm processes sensor measurements in the order they arrive at the fusion center. By updating the fusion result step by step with each incoming measurement, the algorithm enables real-time state estimation. Both the centralized fusion filter and the distributed fusion filter require all measurement data or local estimates to be received before executing the fusion algorithm. When network delays occur during the transmission of local measurements or estimates, both approaches suffer from delayed execution. In particular, in the presence of data packet loss, neither method can function effectively. In contrast, the sequential fusion Kalman filtering algorithm can effectively mitigate the impact of network delays and data loss, enabling timely and robust estimation under such adverse conditions [15].

In practice, for many linear multisensor systems, the process noise is not only correlated with all measurement noises, but the measurement noises themselves are also mutually correlated. Such systems are referred to as Noise Cross-Correlated Systems (NCCSs). One major cause of noise cross-correlation is environmental influence. For example, over the sea surface, the presence of evaporation ducts can cause electromagnetic wave refraction, leading to range or angle measurement errors, which affect the measurement noise. Simultaneously, the evaporation duct may also disturb the airflow around the target, introducing motion perturbations that affect the process noise. Since both the process and measurement noises are driven by the same disturbance source, statistical correlation naturally arises between them [16]. For NCUS, network-induced delays may cause earlier-sampled data to arrive at a later time. When such delayed data are used to perform fusion estimation of the current system state, time alignment is required to construct measurement equations that relate the delayed data to the current state. In these constructed measurement equations, multiple measurement noises may contain shared process noise components due to the system’s dynamics over the delay interval. As a result, statistical correlation is introduced among the measurement noises, thereby transforming the system into NCCS [17,18]. Therefore, given the widespread presence of NCCS, developing a sequential fusion Kalman filter that is equivalent to the centralized fusion Kalman filter holds not only significant scientific importance but also broad application prospects.

For the state fusion estimation problem in NCUS, ref. [19] designed a sequential fusion Kalman filter that is strictly equivalent to a centralized fusion Kalman filter. Through analysis and derivation, the following conditions for this equivalence can be obtained: (1) the noises are mutually independent, which ensures that at each step of sequential fusion, the state estimation error is independent of the newly arriving measurement noise; (2) the innovation sequence of the centralized fusion Kalman filter is equivalent to that of the sequential fusion Kalman filter, i.e., there exists an explicit transformation relationship between the two innovation sequences. For NCCS, we argue that there are two possible approaches to designing a sequential fusion Kalman filter equivalent to the centralized version. The first is to transform the NCCS into an equivalent NCUS, and the second is to convert the correlated innovation sequence of the centralized filter into mutually independent innovation sequences corresponding to a sequential filter bank. In the following, we analyze the non-equivalence between existing sequential fusion Kalman filters and their corresponding centralized fusion Kalman filters along these two lines of thought [20,21,22,23,24,25,26].

For multi-sensor linear systems where process noise and measurement noise are uncorrelated but measurement noise is correlated, refs. [20,21] attempted to eliminate the correlation among measurement noises by equivalently reformulating the measurement equations. Although the rewritten measurement equation is equivalent to the original, the measurement noises in the newly constructed equations remain correlated. Consequently, the state estimation error obtained by the sequential filter also correlates with the new measurement noise. This becomes the reason for the non-equivalence between the designed sequential fusion Kalman filter and the centralized fusion Kalman filter. For NCCS, ref. [22] first augments the system state and then reformulates the sequentially arriving measurement equations using the Gram–Schmidt principle.The system formed by the expanded state equations and the rewritten measurement equations fails to prove equivalence between the new system and the original system. Furthermore, the decorrelation process assumes the variance matrix of process noise is positive definite, whereas it is typically non-negative definite. Therefore, this approach also fails to resolve the equivalence issue with centralized fusion Kalman filters. Ref. [23] uses future state prediction estimates to construct measurement equations for the current state. However, equivalence between the newly established measurement equations and the original ones is difficult to guarantee. The main reason is that the new measurement equations are derived through local filter gain matrix transformations, which are generally non-square and composed of the product of singular and nonsingular matrices, making nonsingularity hard to ensure. Consequently, the sequential fusion Kalman filter designed using the new measurement equation set cannot be considered equivalent to the original centralized fusion Kalman filter.

Ref. [24] attempts to prove the equivalence between the centralized fusion Kalman filter and the sequential fusion Kalman filter by establishing an equivalence relationship between their state update quantities. However, since the information from the centralized fusion filter cannot be equated with that from the sequential fusion filter, and the independence between updates in the sequential filter cannot be guaranteed, the sequential fusion Kalman filter based on this approach is not equivalent to the centralized Kalman filter. Ref. [25] further extends the methodology of [24] to nonlinear multi-sensor systems with noise cross-correlation, demonstrating its applicability in nonlinear cases but still inheriting the same limitations regarding the non-equivalence between sequential and centralized fusion filters. Ref. [26] designed a sequential fusion Kalman filter without modifying the NCCS. Similar to [20,21] the estimation error obtained at each step of sequential fusion is correlated with the noise from newly arriving measurements. The estimation accuracy of the designed sequential fusion Kalman filter is lower than that of the centralized fusion Kalman filter, a conclusion that has also been verified through digital simulations. So far, the state estimation problem for multi-sensor linear systems under noise cross-correlated conditions still lacks effective and comprehensive solutions.

Motivated by the above discussion, this paper proposes a sequential fusion Kalman filter equivalent to a centralized fusion Kalman filter for the state estimation problem of NCCS. The main innovations are as follows: (1) A collaborative decorrelation method was established between noise measurements from a multi-sensor system and prediction estimation errors in state estimation, ingeniously resolving the correlation issue between measurement noise and process noise in the measurement equation. (2) By applying the Gram–Schmidt orthogonalization process to sequentially arriving measurement equations, the correlation between noise components is progressively eliminated, achieving independence between state estimation errors and new measurement noise. (3) Based on the equivalence between the innovations of the two filters, their equivalence is rigorously proven in theory, and simulations further demonstrate the effectiveness of the proposed algorithm. (4) The novel sequential fusion Kalman filter is effectively applicable to state estimation problems under adverse network conditions such as data delay and packet loss, enabling robust and optimized state estimation.

This paper is organized as follows. Section 2 presents the problem formulation. Section 3 introduces the centralized fusion Kalman filter for NCCS. In Section 4, a sequential fusion Kalman filter with stepwise decorrelation for temporally arriving data under noise correlation is proposed. Section 5 provides proof of the equivalence between the designed sequential fusion Kalman filter and the centralized fusion Kalman filter. Section 6 presents simulation examples of a constant-velocity system with three sensors. Finally, conclusions are drawn in Section 7.

Notations: *E* stands for the mathematical expectation; $\delta_{\kappa ,l}$ represents the Kronecker Delta function; the superscripts *T* and −1 denote matrix transpose and matrix inverse, respectively; $R^{n}$ denotes an n-dimensional Euclidean space; diag(∗) denotes a diagonal matrix with ∗ as its diagonal elements; *I* denotes the identity matrix with appropriate dimensions; 0 denotes the zero matrix with with appropriate dimensions;the subscript *i* denotes the *i*th sensor; the superscript (α) denotes the centralized fusion under noise cross-correlation; the superscripts (β) and (γ) enote the sequential fusion and centralized fusion after noise orthogonalization, respectively.

## 2. Problem Statement

Consider a multi-sensor system with *N* sensors described as follows:

(1) $$\begin{array}{c} x(\kappa )=\Phi (\kappa -1)x(\kappa -1)+w(\kappa -1) \end{array}$$

(2) $$\begin{array}{c} z_{i}(\kappa )=H_{i}(\kappa )x\left(\kappa \right)+v_{i}\left(\kappa \right),\;i=1,2,\cdots ,N \end{array}$$

where $x(\kappa )\in R^{n}$ represents the state vector of the system and $z_{i}(\kappa )\in R^{m_{i}}$ is the measurement from the *i*th sensor. Φ(κ−1) is the state transition matrix of the system; w(κ−1) and $v_{i}(\kappa )$ are a zero-mean white Gaussian noise process. $H_{i}(\kappa )$ is the corresponding measurement matrix.

**Assumption 1.**  *The process noise w(κ−1) and measurement noise $v_{i}(\kappa ),i=1,2,\cdots ,N$ are correlated white noises with the following statistical properties.*

(3) $$\begin{array}{c} E\left\{\left(\begin{array}{c} w(\kappa -1)\\ v_{i}(\kappa ) \end{array}\right)\left(\begin{array}{cc} w^{T}(l-1) & v_{j}^{T}(l) \end{array}\right)\right\}=\left[\begin{array}{cc} Q(\kappa -1) & B_{j}(\kappa )\\ B_{j}^{T}(\kappa ) & S_{i,j}(\kappa ) \end{array}\right]\delta_{\kappa ,l} \end{array}$$

*when i=j, $S_{i,j}(\kappa )=R_{i}(\kappa )$.*

**Assumption 2.**  *The initial value x(0) is uncorrelated with w(κ−1) and $v_{i}(\kappa )$, and satisfies*

(4) $$\begin{array}{c} E\left\{x(0)\right\}=x_{0},\;E\left\{\left[x\left(0\right)-x_{0}\right]{\left[x\left(0\right)-x_{0}\right]}^{T}\right\}=P_{0} \end{array}$$

Centralized fusion filters, federated fusion filters, and sequential fusion filters can all be employed to address state estimation problems in multi-sensor systems. The distinction between centralized fusion and sequential fusion has been outlined in the introduction and will not be repeated here. First, federated filtering represents a distributed information fusion approach, offering advantages such as modularity and fault tolerance. However, compared to centralized fusion filters, federated filtering typically yields suboptimal results. Its fusion process relies on estimates from local filters, which fail to adequately account for the correlations among sensor measurements [27]. Second, the overall estimation accuracy heavily depends on the tuning of local filters. Improper selection of local covariance weights can lead to additional accuracy loss, which increases significantly as the number of sensors grows [28].

Therefore, the objective of this paper is to design a novel sequential fusion Kalman filter that is equivalent to the centralized fusion Kalman filter for the multisensor linear system described by Equations (1) and (2) under Assumptions 1 and 2.

## 3. Centralized Fusion Filtering Algorithm (***α***)

To demonstrate the advantages of the proposed algorithm and facilitate comparative analysis, the measurement Equation (2) can be represented in a unified form given by

(5) $$\begin{array}{c} Z(\kappa )=H(\kappa )x(\kappa )+V(\kappa ) \end{array}$$

where

$$\begin{array}{c} Z(\kappa )={\left[z_{1}^{T}(\kappa ),z_{2}^{T}(\kappa ),\cdots ,z_{N}^{T}(\kappa )\right]}^{T} \end{array}$$

$$\begin{array}{c} H(\kappa )={\left[H_{1}^{T}(\kappa ),H_{2}^{T}(\kappa ),\cdots H_{N}^{T}(\kappa )\right]}^{T} \end{array}$$

$$\begin{array}{c} V(\kappa )={\left[v_{1}^{T}(\kappa ),v_{2}^{T}(\kappa ),\cdots ,v_{N}^{T}(\kappa )\right]}^{T} \end{array}$$

The statistical properties of the process noise w(κ−1) and the augmented measurement noise V(κ) are as follows:

(6) $$\begin{array}{c} D(\kappa )=E\left\{w(\kappa -1)V^{T}(\kappa )\right\}=\left[B_{1}(\kappa ),B_{2}(\kappa ),\cdots ,B_{N}(\kappa )\right] \end{array}$$

(7) $$\begin{array}{c} R(\kappa )=E\left\{V(\kappa )V^{T}(\kappa )\right\}=\left[\begin{array}{cccc} R_{1}(\kappa ) & S_{1,2}(\kappa ) & \cdots & S_{1,N}(\kappa )\\ S_{2,1}(\kappa ) & R_{2}(\kappa ) & \cdots & S_{2,N}(\kappa )\\ \vdots & \vdots & \ddots & \vdots \\ S_{N,1}(\kappa ) & S_{N,2}(\kappa ) & \cdots & R_{N`}(\kappa ) \end{array}\right] \end{array}$$

Using the projection theory [29], for the systems (1) and (5), the centralized fusion filter is obtained as follows:

(8) $$\begin{array}{c} \hat{x}(\kappa |\kappa -1)=\Phi (\kappa -1){\hat{x}}^{\left(\alpha \right)}(\kappa -1|\kappa -1) \end{array}$$

(9) $$\begin{array}{cc} \tilde{x}(\kappa |\kappa -1) & =x\left(\kappa \right)-{\hat{x}}^{\left(\alpha \right)}(\kappa |\kappa -1)\\ & =\Phi (\kappa -1){\tilde{x}}^{\left(\alpha \right)}(\kappa -1|\kappa -1)+w(\kappa -1) \end{array}$$

(10) $$\begin{array}{c} P(\kappa |\kappa -1)=\Phi (\kappa -1)P^{\left(\alpha \right)}\left(\kappa -1\right|\kappa -1)\Phi^{T}(\kappa -1)+Q(\kappa -1) \end{array}$$

(11) $$\begin{array}{cc} {\tilde{Z}}^{\left(\alpha \right)}(\kappa |\kappa -1) & =Z\left(\kappa \right)-H\left(\kappa \right)\hat{x}(\kappa |\kappa -1)\\ & =H\left(\kappa \right){\tilde{x}}^{\left(c\right)}(\kappa |\kappa -1)+v\left(\kappa \right) \end{array}$$

(12) $$\begin{array}{cc} P_{\tilde{x}\tilde{Z}}^{\left(\alpha \right)}(\kappa |\kappa -1)\; & =E\left\{{\tilde{x}}^{\left(\alpha \right)}\left(\kappa |\kappa -1\right){\tilde{Z}}^{(\alpha )T}\left(\kappa |\kappa -1\right)\right\}\\ \; & =P(\kappa |\kappa -1)H^{T}\left(\kappa \right)+D\left(\kappa \right) \end{array}$$

(13) $$\begin{array}{cc} P_{\tilde{Z}\tilde{Z}}^{\left(\alpha \right)}(\kappa |\kappa -1)\; & =E\left\{{\tilde{Z}}^{\left(\alpha \right)}\left(\kappa |\kappa -1\right){\tilde{Z}}^{(\alpha )T}\left(\kappa |\kappa -1\right)\right\}\\ \; & =H\left(\kappa \right)P(\kappa |\kappa -1)H^{T}\left(\kappa \right)+H\left(\kappa \right)D\left(\kappa \right)\\ \; & +D^{T}\left(\kappa \right)H^{T}\left(\kappa \right)+R\left(\kappa \right) \end{array}$$

(14) $$\begin{array}{c} K^{\left(\alpha \right)}\left(\kappa \right)=P_{\tilde{x}\tilde{Z}}^{\left(\alpha \right)}(\kappa |\kappa -1)P_{\tilde{Z}\tilde{Z}}^{{\left(\alpha \right)}^{-1}}\left(\kappa \right|\kappa -1) \end{array}$$

(15) $$\begin{array}{c} {\hat{x}}^{\left(\alpha \right)}\left(\kappa |\kappa \right)=\hat{x}(\kappa |\kappa -1)+K^{\left(\alpha \right)}\left(\kappa \right){\tilde{Z}}^{\left(\alpha \right)}(\kappa |\kappa -1) \end{array}$$

(16) $$\begin{array}{c} P^{\left(\alpha \right)}\left(\kappa |\kappa \right)=P(\kappa |\kappa -1)-K^{\left(\alpha \right)}\left(\kappa \right)P_{ZZ}^{\left(\alpha \right)}\left(\kappa \right|\kappa -1)K^{(\alpha )T}\left(\kappa \right) \end{array}$$

where $\hat{x}\left(\kappa |\kappa -1\right)$ and $\tilde{x}\left(\kappa |\kappa -1\right)$ denote the predicted state estimate and the predicted estimation error covariance, respectively; ${\tilde{Z}}^{\left(\alpha \right)}\left(\kappa |\kappa -1\right)$ s the innovation vector with covariance matrix $P_{\tilde{Z}\tilde{Z}}^{\left(\alpha \right)}\left(\kappa \right|\kappa -1)$; $P_{\tilde{x}\tilde{Z}}^{\left(\alpha \right)}\left(\kappa \right|\kappa -1)$ is the cross-covariance matrix between the state prediction error $\tilde{x}\left(\kappa |\kappa -1\right)$ and the innovation ${\tilde{Z}}^{\left(\alpha \right)}\left(\kappa |\kappa -1\right)$; P(κ|κ−1) and $K^{\left(\alpha \right)}\left(\kappa \right)$ denote the predicted estimation error covariance matrix and the filtering gain matrix, respectively; ${\hat{x}}^{\left(\alpha \right)}\left(\kappa |\kappa \right)$ and $P^{\left(\alpha \right)}\left(\kappa |\kappa \right)$ denote the centralized filter estimate and the estimation error covariance matrix, respectively.

**Remark 1.**  *Equations (8)–(16) constitute the centralized fusion Kalman filter that accounts for noise cross-correlation. Neglecting the correlation between the process noise and the measurement noise by setting D(κ)=0 leads to increased estimation error and degraded filtering performance. This observation will be confirmed through the simulation results presented later.*

**Remark 2.**  *In the case of simultaneous arrival of multi-sensor data, the centralized fusion Kalman filter provides an effective optimal filtering solution under noise correlation. Nevertheless, it requires high-dimensional matrix inversions, leading to substantial computational complexity. In multi-sensor network environments, measurement data acquired at the same time by spatially distributed sensors are often subject to transmission delays and network instability, making it difficult for the data to arrive at the fusion center simultaneously. However, the centralized fusion Kalman filter requires delaying the filtering process until the latest arriving sensor data is received. Furthermore, in cases where data from certain sensors are lost due to network failures, the centralized approach may become inapplicable or fail to execute. Therefore, it is necessary to design a sequential fusion Kalman filter that is equivalent to the centralized fusion Kalman filter, in order to address the problem of real-time state estimation under network delays and data packet loss.*

## 4. Sequential Fusion Filtering Algorithm (*β*)

Since the measurement equation sets arriving in different orders are related to the naturally ordered set by a nonsingular elementary transformation matrix, they are therefore equivalent. Without loss of generality, this section assumes that the measurements from the sensors at the same time step arrive in their natural order, i.e., $z_{1},z_{2},\cdots ,z_{N}$.

First, a one-step ahead prediction of the state is performed and regarded as a measurement of the state, thereby establishing the 0-th measurement equation, where the measurement noise corresponds to the one-step prediction error. Then, the first arriving measurement equation is reformulated using the Gram–Schmidt orthogonalization principle. The reformulated measurement equation is treated as the new first measurement equation, such that the new measurement noise is uncorrelated with the measurement noise in the 0-th equation, i.e., the state prediction error. By analogy, all subsequent measurement equations are sequentially reformulated using the same principle, ensuring that in the resulting new measurement equations, the new measurement noises are not only uncorrelated with the process noise but also mutually uncorrelated. Based on these reformulated equations, a novel sequential fusion Kalman filter is constructed.

### 4.1. State Estimation Based on Measurement Equation (1)

To begin with, the state prediction Equation (8) can be equivalently reformulated as a measurement equation for the state x(κ). For convenience of exposition, this is referred to as the 0-th measurement equation of x(κ).

(17) $$\begin{array}{c} {\bar{z}}_{0}\left(\kappa \right)={\bar{H}}_{0}\left(\kappa \right)x\left(\kappa \right)+{\bar{v}}_{0}\left(\kappa \right) \end{array}$$

where ${\bar{z}}_{0}\left(\kappa \right)=\hat{x}\left(\kappa |\kappa -1\right)$, ${\bar{H}}_{0}\left(\kappa \right)=I$, and ${\bar{v}}_{0}\left(\kappa \right)=-\tilde{x}\left(\kappa |\kappa -1\right)$. Since the prediction error is a zero-mean random variable, its covariance matrix is P(κ|κ−1). At this point, the statistical properties of ${\bar{v}}_{0}\left(\kappa \right)$ satisfy ${\bar{v}}_{0}\left(\kappa \right)∼\;N\left[0,{\bar{R}}_{0}\left(\kappa \right)\right],{\bar{R}}_{0}\left(\kappa \right)=P\left(\kappa |\kappa -1\right)$.

**Remark 3.**  *Equation (17) equivalently represents the one-step-ahead prediction equation as a measurement equation for the state x(κ). This equivalent representation formally unifies the prediction value and sensor measurements into the same category of measurement equations, facilitating uniform decorrelation processing for all noise terms (including prediction errors and measurement noise) during the subsequent Gram–Schmidt orthogonalization process.*

(1) Equivalently reformulate measurement Equation (1)

(18) $$\begin{array}{cc} z_{1}\left(\kappa \right) & =H_{1}\left(\kappa \right)x\left(\kappa \right)+v_{1}\left(\kappa \right)\\ & +G_{1,0}\left(\kappa \right)\left[{\bar{z}}_{0}(\kappa )-{\bar{H}}_{0}(\kappa )x(\kappa )-{\bar{v}}_{0}(\kappa )\right] \end{array}$$

After combining like terms, we obtain

(19) $$\begin{array}{c} {\bar{z}}_{1}\left(\kappa \right)={\bar{H}}_{1}\left(\kappa \right)x\left(\kappa \right)+{\bar{v}}_{1}\left(\kappa \right) \end{array}$$

where ${\bar{z}}_{1}\left(\kappa \right)=z_{1}\left(\kappa \right)-G_{1,0}\left(\kappa \right){\bar{z}}_{0}\left(\kappa \right)$, ${\bar{H}}_{1}\left(\kappa \right)=H_{1}\left(\kappa \right)-G_{1,0}\left(\kappa \right){\bar{H}}_{0}\left(\kappa \right)$, ${\bar{v}}_{1}\left(\kappa \right)=v_{1}\left(\kappa \right)-G_{1,0}\left(\kappa \right){\bar{v}}_{0}\left(\kappa \right)$.

(2) Based on the conditions required by the Gram–Schmidt orthogonalization principle, $E\left\{{\bar{v}}_{1}\left(\kappa \right){\bar{v}}_{0}^{T}\left(\kappa \right)\right\}=0$ is obtained, and the decorrelation matrix $G_{1,0}\left(\kappa \right)$ is derived as follows

(20) $$\begin{array}{c} G_{1,0}\left(\kappa \right)=F_{1,0}\left(\kappa \right){\bar{R}}_{0}^{-1}\left(\kappa \right) \end{array}$$

where

$$\begin{array}{cc} F_{1,0}\left(\kappa \right) & =E\left\{v_{1}(\kappa ){\bar{v}}_{0}^{T}(\kappa )\right\}\\ & =-E\left\{v_{1}(\kappa ){\tilde{x}}^{T}\left(\kappa |\kappa -1\right)\right\}\\ & =-E\left\{v_{1}(\kappa )w^{T}\left(\kappa -1\right)\right\}\\ & =-B_{1}^{T}\left(\kappa \right) \end{array}$$

The definition of $F_{1,0}\left(\kappa \right)$ originates from the cross-correlation definition of process noise and measurement noise in Assumption 1. In addition, we have ${\bar{v}}_{1}\left(\kappa \right)∼\;N\left[0,{\bar{R}}_{1}\left(\kappa \right)\right]$, ${\bar{R}}_{1}\left(\kappa \right)$ calculated as follows:

(21) $$\begin{array}{cc} {\bar{R}}_{1}\left(\kappa \right) & =E\left\{{\bar{v}}_{1}(\kappa ){\bar{v}}_{1}^{T}\left(\kappa \right)\right\}\\ & =R_{1}\left(\kappa \right)-F_{1,0}\left(\kappa \right){\bar{R}}_{0}^{-1}\left(\kappa \right)F_{1,0}^{T}\left(\kappa \right) \end{array}$$

(3) Based on the state Equation (1) and measurement Equation (19), the corresponding one-step sequential Kalman filter is derived as follows:

(22) $$\begin{array}{c} {\hat{\bar{x}}}_{1}\left(\kappa |\kappa \right)=\hat{x}\left(\kappa |\kappa -1\right)+{\bar{K}}_{1}\left(\kappa \right){\tilde{\bar{z}}}_{1}\left(\kappa |\kappa -1\right) \end{array}$$

(23) $$\begin{array}{cc} {\bar{P}}_{1}\left(\kappa |\kappa \right)\; & =E\left\{{\tilde{\bar{x}}}_{1}\left(\kappa |\kappa \right){\tilde{\bar{x}}}_{1}^{T}\left(\kappa |\kappa \right)\right\}\\ \; & =P\left(\kappa |\kappa -1\right)-{\bar{K}}_{1}\left(\kappa \right){\bar{P}}_{{\tilde{\bar{z}}}_{1}{\tilde{\bar{z}}}_{1}}\left(\kappa |\kappa -1\right){\bar{K}}_{1}^{T}\left(\kappa \right) \end{array}$$

where

$$\begin{array}{c} {\bar{K}}_{1}\left(\kappa \right)={\bar{P}}_{\tilde{x}{\tilde{\bar{z}}}_{1}}(\kappa |\kappa -1){\bar{P}}_{{\tilde{\bar{z}}}_{1}{\tilde{\bar{z}}}_{1}}^{-1}\left(\kappa \right|\kappa -1) \end{array}$$

$$\begin{array}{c} {\tilde{\bar{z}}}_{1}\left(\kappa |\kappa -1\right)={\bar{z}}_{1}\left(\kappa |\kappa -1\right)-{\bar{H}}_{1}\left(\kappa \right)\hat{x}\left(\kappa |\kappa -1\right) \end{array}$$

$$\begin{array}{c} {\bar{P}}_{\tilde{x}{\tilde{\bar{z}}}_{1}}(\kappa |\kappa -1)=E\left\{\tilde{x}\left(\kappa |\kappa -1\right){\tilde{\bar{z}}}_{1}^{T}\left(\kappa |\kappa -1\right)\right\}=P\left(\kappa \right|\kappa -1){\bar{H}}_{1}^{T}\left(\kappa \right) \end{array}$$

$$\begin{array}{cc} {\bar{P}}_{{\tilde{\bar{z}}}_{1}{\tilde{\bar{z}}}_{1}}\left(\kappa |\kappa -1\right)\; & =E\left\{{\tilde{\bar{z}}}_{1}\left(\kappa |\kappa -1\right){\tilde{\bar{z}}}_{1}^{T}\left(\kappa |\kappa -1\right)\right\}\\ \; & ={\bar{H}}_{1}\left(\kappa \right)P\left(\kappa |\kappa -1\right){\bar{H}}_{1}^{T}\left(\kappa \right)+{\bar{R}}_{1}\left(\kappa \right) \end{array}$$

$$\begin{array}{c} {\tilde{\bar{x}}}_{1}\left(\kappa |\kappa \right)=x\left(\kappa \right)-{\hat{\bar{x}}}_{1}\left(\kappa |\kappa \right)=\tilde{x}\left(\kappa |\kappa -1\right)-{\bar{K}}_{1}\left(\kappa \right){\tilde{\bar{z}}}_{1}\left(\kappa \right) \end{array}$$

### 4.2. Sequential Fusion Filter for the State Based on Measurement Equations (1) and (2)

According to the concept of sequential filtering, the initial conditions are given by ${\hat{\bar{x}}}_{2}\left(\kappa |\kappa -1\right)={\hat{\bar{x}}}_{1}\left(\kappa |\kappa \right)$, ${\tilde{\bar{x}}}_{2}\left(\kappa |\kappa -1\right)={\tilde{\bar{x}}}_{1}\left(\kappa |\kappa \right)$, and ${\bar{P}}_{2}\left(\kappa |\kappa -1\right)={\bar{P}}_{1}\left(\kappa |\kappa \right)$.

(1) Equivalently reformulate measurement Equation (2)

(24) $$\begin{array}{cc} z_{2}\left(\kappa \right)\; & =H_{2}\left(\kappa \right)x\left(\kappa \right)+v_{2}\left(\kappa \right)\\ \; & +G_{2,0}\left(\kappa \right)\left[{\bar{z}}_{0}\left(\kappa \right)-{\bar{H}}_{0}\left(\kappa \right)x\left(\kappa \right)-{\bar{v}}_{0}\left(\kappa \right)\right]\\ \; & +G_{2,1}\left(\kappa \right)\left[{\bar{z}}_{1}\left(\kappa \right)-{\bar{H}}_{1}\left(\kappa \right)x\left(\kappa \right)-{\bar{v}}_{1}\left(\kappa \right)\right] \end{array}$$

After combining like terms, we obtain

(25) $$\begin{array}{c} {\bar{z}}_{2}\left(\kappa \right)={\bar{H}}_{2}\left(\kappa \right)x\left(\kappa \right)+{\bar{v}}_{2}\left(\kappa \right) \end{array}$$

where

$$\begin{array}{c} {\bar{z}}_{2}\left(\kappa \right)=z_{2}\left(\kappa \right)-G_{2,0}\left(\kappa \right){\bar{z}}_{0}\left(\kappa \right)-G_{2,1}\left(\kappa \right){\bar{z}}_{1}\left(\kappa \right) \end{array}$$

$$\begin{array}{c} {\bar{H}}_{2}\left(\kappa \right)=H_{2}\left(\kappa \right)-G_{2,0}\left(\kappa \right){\bar{H}}_{0}\left(\kappa \right)-G_{2,1}\left(\kappa \right){\bar{H}}_{1}\left(\kappa \right) \end{array}$$

$$\begin{array}{c} {\bar{v}}_{2}\left(\kappa \right)=v_{2}\left(\kappa \right)-G_{2,0}\left(\kappa \right){\bar{v}}_{0}\left(\kappa \right)-G_{2,1}\left(\kappa \right){\bar{v}}_{1}\left(\kappa \right) \end{array}$$

(2) Based on the conditions required by the Gram–Schmidt orthogonalization principle, $E\left\{{\bar{v}}_{2}\left(\kappa \right){\bar{v}}_{0}^{T}\left(\kappa \right)\right\}=0$ and $E\left\{{\bar{v}}_{2}\left(\kappa \right){\bar{v}}_{1}^{T}\left(\kappa \right)\right\}=0$ are obtained, and the decorrelation matrix $G_{2,0}\left(\kappa \right)$ and $G_{2,1}\left(\kappa \right)$ are derived as follows

(26) $$\begin{array}{c} G_{2,0}\left(\kappa \right)=F_{2,0}\left(\kappa \right){\bar{R}}_{0}^{-1}\left(\kappa \right) \end{array}$$

(27) $$\begin{array}{c} G_{2,1}\left(\kappa \right)=F_{2,1}\left(\kappa \right){\bar{R}}_{1}^{-1}\left(\kappa \right) \end{array}$$

where

$$\begin{array}{cc} F_{2,0}\left(\kappa \right) & =E\left\{v_{2}\left(\kappa \right)\;{\bar{v}}_{0}^{T}\left(\kappa \right)\right\}\\ & =-B_{2}^{T}\left(\kappa \right) \end{array}$$

$$\begin{array}{cc} F_{2,1}\left(\kappa \right)\; & =E\left\{v_{2}\left(\kappa \right){\bar{v}}_{1}^{T}\left(\kappa \right)\right\}\\ & =E\left\{v_{2}\left(\kappa \right){\left[v_{1}\left(\kappa \right)-G_{1,0}\left(\kappa \right){\bar{v}}_{0}\left(\kappa \right)\right]}^{T}\right\}\\ & =S_{2,1}\left(\kappa \right)+B_{2}^{T}\left(\kappa \right)G_{1,0}^{T}\left(\kappa \right) \end{array}$$

The measurement noise ${\bar{v}}_{2}\left(\kappa \right)$ satisfies ${\bar{v}}_{2}\left(\kappa \right)∼\;N\left[0,{\bar{R}}_{2}\left(\kappa \right)\right]$, ${\bar{R}}_{2}\left(\kappa \right)$ is calculated as follow:

(28) $$\begin{array}{cc} {\bar{R}}_{2}\left(\kappa \right)\; & =E\left\{{\bar{v}}_{2}\left(\kappa \right){\bar{v}}_{2}^{T}\left(\kappa \right)\right\}\\ \; & =R_{2}\left(\kappa \right)-F_{2,0}\left(\kappa \right){\bar{R}}_{0}^{-1}\left(\kappa \right)F_{2,0}^{T}\left(\kappa \right)-F_{2,1}\left(\kappa \right){\bar{R}}_{1}^{-1}\left(\kappa \right)F_{2,1}^{T}\left(\kappa \right) \end{array}$$

(3) Based on the state equation and the measurement Equation (25), the two-step sequential fusion Kalman filter is established as follows:

(29) $$\begin{array}{c} {\hat{\bar{x}}}_{2}\left(\kappa |\kappa \right)={\hat{\bar{x}}}_{1}\left(\kappa |\kappa \right)+{\bar{K}}_{2}\left(\kappa \right){\tilde{\bar{z}}}_{2}\left(\kappa |\kappa -1\right) \end{array}$$

(30) $$\begin{array}{cc} {\bar{P}}_{2}\left(\kappa |\kappa \right)\; & =E\left\{{\tilde{\bar{x}}}_{2}\left(\kappa |\kappa \right)\;{\tilde{\bar{x}}}_{2}^{T}\left(\kappa |\kappa \right)\right\}\\ & ={\bar{P}}_{1}\left(\kappa |\kappa \right)-{\bar{K}}_{2}\left(\kappa \right)\;{\bar{P}}_{{\tilde{\bar{z}}}_{2}{\tilde{\bar{z}}}_{2}}\left(\kappa |\kappa -1\right)\;{\bar{K}}_{2}^{T}\left(\kappa \right) \end{array}$$

where

$$\begin{array}{c} {\bar{K}}_{2}\left(\kappa \right)={\bar{P}}_{{\tilde{\bar{x}}}_{1}{\tilde{\bar{z}}}_{2}}(\kappa |\kappa -1){\bar{P}}_{{\tilde{\bar{z}}}_{2}{\tilde{\bar{z}}}_{2}}^{-1}\left(\kappa \right|\kappa -1) \end{array}$$

$$\begin{array}{c} {\tilde{\bar{z}}}_{2}\left(\kappa |\kappa -1\right)={\bar{z}}_{2}\left(\kappa |\kappa -1\right)-{\bar{H}}_{2}\left(\kappa \right){\hat{\bar{x}}}_{2}\left(\kappa |\kappa \right) \end{array}$$

$$\begin{array}{c} {\bar{P}}_{{\tilde{\bar{x}}}_{1}{\tilde{\bar{z}}}_{2}}(\kappa |\kappa -1)=E\left\{{\tilde{\bar{x}}}_{1}\left(\kappa |\kappa -1\right){\tilde{\bar{z}}}_{2}^{T}\left(\kappa |\kappa -1\right)\right\}={\bar{P}}_{1}\left(\kappa \right|\kappa ){\bar{H}}_{2}^{T}\left(\kappa \right) \end{array}$$

$$\begin{array}{cc} {\bar{P}}_{{\tilde{\bar{z}}}_{2}{\tilde{\bar{z}}}_{2}}\left(\kappa |\kappa -1\right)\; & =E\left\{{\tilde{\bar{z}}}_{2}\left(\kappa |\kappa -1\right)\;{\tilde{\bar{z}}}_{2}^{T}\left(\kappa |\kappa -1\right)\right\}\\ \; & ={\bar{H}}_{2}\left(\kappa \right)\;{\bar{P}}_{1}\left(\kappa |\kappa \right)\;{\bar{H}}_{2}^{T}\left(\kappa \right)+{\bar{R}}_{2}\left(\kappa \right) \end{array}$$

$$\begin{array}{c} {\tilde{\bar{x}}}_{2}\left(\kappa |\kappa \right)=x\left(\kappa \right)-{\hat{\bar{x}}}_{2}\left(\kappa |\kappa \right)={\tilde{\bar{x}}}_{1}\left(\kappa |\kappa \right)-{\bar{K}}_{2}\left(\kappa \right){\tilde{\bar{z}}}_{2}\left(\kappa \right) \end{array}$$

### 4.3. Sequential Fusion Filter for the State Based on Measurement Equations from i−1 to *i*

Assuming that the measurement equations up to step i−1 have been orthogonalized via the Gram–Schmidt process, the transformed measurement equation of the i−1-th sensor is ${\bar{z}}_{i-1}\left(\kappa \right)={\bar{H}}_{i-1}\left(\kappa \right)x\left(\kappa \right)+{\bar{v}}_{i-1}\left(\kappa \right)$. The corresponding i−1-th step of the sequential fusion Kalman filter is then given as follows:

(31) $$\begin{array}{cc} {\hat{\bar{x}}}_{i-1}\left(\kappa |\kappa \right)\; & =E\;\left\{x\left(\kappa \right)\;|\;{\hat{x}}_{0},\;{\bar{z}}_{1}\left(\kappa \right),\;\cdots ,\;{\bar{z}}_{i-1}\left(\kappa \right)\right\}\\ \; & =E\;\left\{x\left(\kappa \right)\;|\;{\hat{\bar{x}}}_{i-2}\left(\kappa |\kappa \right),\;{\bar{z}}_{i-1}\left(\kappa \right)\right\} \end{array}$$

(32) $$\begin{array}{c} {\tilde{\bar{x}}}_{i-1}\left(\kappa |\kappa \right)=x\left(\kappa \right)-{\hat{\bar{x}}}_{i-1}\left(\kappa |\kappa \right) \end{array}$$

(33) $$\begin{array}{c} {\bar{P}}_{i-1}\left(\kappa |\kappa \right)=E\left\{{\tilde{\bar{x}}}_{i-1}\left(\kappa |\kappa \right){\tilde{\bar{x}}}_{i-1}^{T}\left(\kappa |\kappa \right)\right\} \end{array}$$

This section aims to establish a recursive sequential fusion Kalman filter by equivalently transforming the measurement equation via the Gram–Schmidt orthogonalization after the *i*-th sensor measurement becomes available.

(1) Equivalently reformulate measurement equation *i*

(34) $$\begin{array}{cc} z_{i}\left(\kappa \right)\; & =H_{i}\left(\kappa \right)x\left(\kappa \right)+v_{i}\left(\kappa \right)\\ \; & \;+G_{i,0}\left(\kappa \right)\left[{\bar{z}}_{0}\left(\kappa \right)-{\bar{H}}_{0}\left(\kappa \right)x\left(\kappa \right)-{\bar{v}}_{0}\left(\kappa \right)\right]\\ \; & \;+\sum_{j=1}^{i-1}G_{i,j}\left(\kappa \right)\left[{\bar{z}}_{j}\left(\kappa \right)-{\bar{H}}_{j}\left(\kappa \right)x\left(\kappa \right)-{\bar{v}}_{j}\left(\kappa \right)\right] \end{array}$$

After combining like terms, we obtain

(35) $$\begin{array}{c} {\bar{z}}_{i}\left(\kappa \right)={\bar{H}}_{i}\left(\kappa \right)x\left(\kappa \right)+{\bar{v}}_{i}\left(\kappa \right),i=1,2,\cdots ,N \end{array}$$

where

(36) $$\begin{array}{c} {\bar{z}}_{i}\left(\kappa \right)=z_{i}\left(\kappa \right)-G_{i,0}\left(\kappa \right){\bar{z}}_{0}\left(\kappa \right)-\sum_{j=1}^{i-1}G_{i,j}\left(\kappa \right){\bar{z}}_{j}\left(\kappa \right) \end{array}$$

(37) $$\begin{array}{c} {\bar{H}}_{i}\left(\kappa \right)=H_{i}\left(\kappa \right)-G_{i,0}\left(\kappa \right){\bar{H}}_{0}\left(\kappa \right)-\sum_{j=1}^{i-1}G_{i,j}\left(\kappa \right){\bar{H}}_{j}\left(\kappa \right) \end{array}$$

(38) $$\begin{array}{c} {\bar{v}}_{i}\left(\kappa \right)=v_{i}\left(\kappa \right)-G_{i,0}\left(\kappa \right){\bar{v}}_{0}\left(\kappa \right)-\sum_{j=1}^{i-1}G_{i,j}\left(\kappa \right){\bar{v}}_{j}\left(\kappa \right) \end{array}$$

(2) Based on the conditions required by the Gram–Schmidt orthogonalization principle, $E\left\{{\bar{v}}_{i}\left(\kappa \right){\bar{v}}_{0}^{T}\left(\kappa \right)\right\}=0$ and $E\left\{{\bar{v}}_{i}\left(\kappa \right){\bar{v}}_{j}^{T}\left(\kappa \right)\right\}=0,j=1,2,\cdots ,i-1$ are obtained, and the decorrelation matrix $G_{i,0}\left(\kappa \right)$ and $G_{i}\left(\kappa \right)$ are derived as follows

(39) $$\begin{array}{c} G_{i,0}\left(\kappa \right)=F_{i,0}\left(\kappa \right){\bar{R}}_{0}^{-1}\left(\kappa \right) \end{array}$$

(40) $$\begin{array}{c} G_{i}\left(\kappa \right)=F_{i}\left(\kappa \right){\bar{R}}_{i}^{-1}\left(\kappa \right) \end{array}$$

where

(41) $$\begin{array}{c} \left\{\begin{array}{c} F_{i,0}(\kappa )=E\left\{v_{i}\left(\kappa \right){\bar{v}}_{0}^{T}\left(\kappa \right)\right\}=-B_{i}^{T}(\kappa )\\ F_{i}(\kappa )=E\left\{v_{i}\left(\kappa \right)\left[{\bar{v}}_{1}^{T}\left(\kappa \right),{\bar{v}}_{2}^{T}\left(\kappa \right),\cdots ,{\bar{v}}_{i-1}^{T}\left(\kappa \right)\right]\right\}\\ \;\;=diag(F_{i,1}\left(k\right),F_{i,2}\left(k\right),\cdots ,F_{i,i-1}\left(k\right)) \end{array}\right. \end{array}$$

(42) $$\begin{array}{c} \left\{\begin{array}{c} G_{i,0}(\kappa )=F_{i,0}(\kappa )R_{0}^{-1}(\kappa )\\ G_{i}(\kappa )=diag\left(G_{i,1}\left(\kappa \right),G_{i,2}\left(\kappa \right),\cdots ,G_{i,i-1}\left(\kappa \right)\right)=F_{i}(\kappa ){\bar{R}}_{i-1}^{-1}(\kappa ) \end{array}\right. \end{array}$$

Moreover, the correlation matrix $F_{i}\left(\kappa \right)$ and the variance ${\bar{R}}_{i}\left(\kappa \right)$ are given as follows:

(43) $$\begin{array}{c} \left\{\begin{array}{c} F_{2,1}(\kappa )=E\left\{v_{2}\left(\kappa \right){\bar{v}}_{1}^{T}\left(\kappa \right)\right\}=S_{2,1}(\kappa )+B_{2}^{T}(\kappa )G_{1,0}^{T}(\kappa )\\ \vdots \\ F_{i,i-1}(\kappa )=E\left\{v_{i}\left(\kappa \right){\bar{v}}_{i-1}^{T}\left(\kappa \right)\right\}\\ \;\;=S_{i,i-1}(\kappa )+B_{i}^{T}(\kappa )G_{i-1,0}^{T}(\kappa )-\sum_{j=1}^{i-2}F_{i,j}(\kappa )G_{i-1,j}^{T}(\kappa ) \end{array}\right. \end{array}$$

(44) $$\begin{array}{c} \begin{array}{c} {\bar{R}}_{i}\left(\kappa \right)=E\left\{{\bar{v}}_{i}\left(\kappa \right){\bar{v}}_{i}^{T}\left(\kappa \right)\right\}\\ =\left\{\begin{array}{c} R_{1}\left(\kappa \right)-F_{1,0}\left(\kappa \right)R_{0}^{-1}\left(\kappa \right)F_{1,0}^{T}\left(\kappa \right),i=1\\ R_{i}\left(\kappa \right)-F_{i,0}\left(\kappa \right)R_{0}^{-1}\left(\kappa \right)F_{i,0}^{T}\left(\kappa \right)-F_{i}\left(\kappa \right){\bar{R}}_{i-1}^{-1}\left(\kappa \right)F_{i}^{T}\left(\kappa \right),i=2,3,\cdots ,N \end{array}\right. \end{array} \end{array}$$

(3) Based on the state equation and the measurement Equation (35), the *i* sequential fusion Kalman filter is established as follows:

(45) $$\begin{array}{c} {\hat{\bar{x}}}_{i}\left(\kappa |\kappa \right)={\hat{\bar{x}}}_{i-1}\left(\kappa |\kappa \right)+{\bar{K}}_{i}\left(\kappa \right){\tilde{\bar{z}}}_{i}\left(\kappa |\kappa -1\right) \end{array}$$

(46) $$\begin{array}{cc} {\bar{P}}_{i}\left(\kappa |\kappa \right) & =E\left\{{\tilde{\bar{x}}}_{i}\left(\kappa |\kappa \right){\tilde{\bar{x}}}_{i}^{T}\left(\kappa |\kappa \right)\right\}\\ \; & ={\bar{P}}_{i-1}\left(\kappa |\kappa \right)-{\bar{K}}_{i}\left(\kappa \right){\bar{P}}_{{\tilde{\bar{z}}}_{i}{\tilde{\bar{z}}}_{i}}\left(\kappa |\kappa -1\right){\bar{K}}_{i}^{T}\left(\kappa \right) \end{array}$$

where

(47) $$\begin{array}{c} {\bar{K}}_{i}\left(\kappa \right)={\bar{P}}_{{\tilde{\bar{x}}}_{i-1}{\tilde{\bar{z}}}_{i}}(\kappa |\kappa -1){\bar{P}}_{{\tilde{\bar{z}}}_{i}{\tilde{\bar{z}}}_{i}}^{-1}\left(\kappa \right|\kappa -1) \end{array}$$

(48) $$\begin{array}{c} {\tilde{\bar{z}}}_{i}\left(\kappa |\kappa -1\right)={\bar{z}}_{i}\left(\kappa |\kappa -1\right)-{\bar{H}}_{i}\left(\kappa \right){\hat{\bar{x}}}_{i}\left(\kappa |\kappa -1\right) \end{array}$$

(49) $$\begin{array}{c} {\bar{P}}_{{\tilde{\bar{x}}}_{i-1}{\tilde{\bar{z}}}_{i}}(\kappa |\kappa -1)=E\left\{{\tilde{\bar{z}}}_{i}\left(\kappa |\kappa -1\right){\tilde{\bar{z}}}_{i}^{T}\left(\kappa |\kappa -1\right)\right\}={\bar{P}}_{i-1}\left(\kappa \right|\kappa ){\bar{H}}_{i}^{T}\left(\kappa \right) \end{array}$$

(50) $$\begin{array}{c} {\bar{P}}_{{\tilde{\bar{z}}}_{i}{\tilde{\bar{z}}}_{i}}(\kappa |\kappa -1)={\bar{H}}_{i}\left(\kappa \right){\bar{P}}_{i-1}\left(\kappa \right|\kappa ){\bar{H}}_{i}^{T}\left(\kappa \right)+{\bar{R}}_{i}\left(\kappa \right) \end{array}$$

(51) $$\begin{array}{c} {\tilde{\bar{x}}}_{i}\left(\kappa |\kappa \right)=x\left(\kappa \right)-{\hat{\bar{x}}}_{i-1}\left(\kappa |\kappa \right)={\tilde{\bar{x}}}_{i-1}\left(\kappa |\kappa \right)-{\bar{K}}_{i}\left(\kappa \right){\tilde{\bar{z}}}_{i}\left(\kappa \right) \end{array}$$

The globally optimal sequential fusion estimator ${\hat{\bar{x}}}_{N}\left(\kappa |\kappa \right)$ of the state x(κ), and its covariance matrix ${\bar{P}}_{N}\left(\kappa |\kappa \right)$ can be obtained when i=N.

(52) $$\begin{array}{c} {\hat{\bar{x}}}^{\left(\beta \right)}\left(\kappa |\kappa \right)={\hat{\bar{x}}}_{N}\left(\kappa |\kappa \right),{\bar{P}}^{\left(\beta \right)}\left(\kappa |\kappa \right)={\bar{P}}_{N}\left(\kappa |\kappa \right) \end{array}$$

By repeatedly iterating Equations (45), (46) and (51), an alternative formulation of the sequential fusion Kalman filter (β) as

(53) $$\begin{array}{c} {\hat{\bar{x}}}^{\left(\beta \right)}\left(\kappa |\kappa \right)=\hat{x}\left(\kappa |\kappa -1\right)+\sum_{l=1}^{N}{\bar{K}}_{l}\left(\kappa \right){\tilde{\bar{z}}}_{l}\left(\kappa |\kappa -1\right) \end{array}$$

(54) $$\begin{array}{c} {\tilde{\bar{x}}}^{\left(\beta \right)}\left(\kappa |\kappa \right)=\tilde{x}\left(\kappa |\kappa -1\right)-\sum_{l=1}^{N}{\bar{K}}_{l}\left(\kappa \right){\tilde{\bar{z}}}_{l}\left(\kappa |\kappa -1\right) \end{array}$$

(55) $$\begin{array}{c} {\bar{P}}^{\left(\beta \right)}\left(\kappa |\kappa \right)=P\left(\kappa |\kappa -1\right)-\sum_{l=1}^{N}{\bar{K}}_{l}\left(\kappa \right){\bar{P}}_{{\tilde{\bar{z}}}_{l}{\tilde{\bar{z}}}_{l}}\left(\kappa |\kappa -1\right){\bar{K}}_{l}^{T}\left(\kappa \right) \end{array}$$

**Figure 1:**
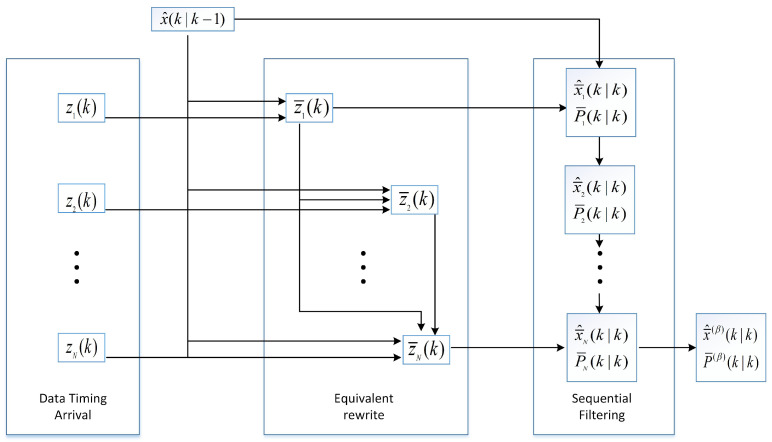
Algorithm flowchart of the sequential algorithm.

Figure 1 illustrates the flowchart of the sequential fusion Kalman filtering algorithm, in which noise correlations are eliminated progressively at each step.

**Remark 4.**  *According to the Gram–Schmidt orthogonalization principle, the measurement noises ${\bar{v}}_{i}\left(\kappa \right)$ in Equation (35) are mutually uncorrelated and also uncorrelated with ${\bar{v}}_{0}\left(\kappa \right)$. Within the framework of the sequential filtering method, the fusion center can then use the equivalent measurement ${\bar{z}}_{i}\left(\kappa \right)$ of $z_{i}\left(\kappa \right)$ to further update the state estimate at time κ.*

## 5. Equivalence Analysis of Filters

To facilitate the analysis of the equivalence among the filters, this subsection first designs a centralized fusion Kalman filter based on the state Equation (1) and the new measurement Equation (35), denoted as Filter (γ). This filter serves as an intermediate reference to prove the equivalence between Filter (β) and Filter (α).

### 5.1. Centralized Fusion Filtering Algorithm (γ)

A unified representation of the measurement Equation (36)

(56) $$\begin{array}{c} \bar{Z}\left(\kappa \right)=\bar{H}\left(\kappa \right)x\left(\kappa \right)+\bar{V}\left(\kappa \right) \end{array}$$

where

$$\begin{array}{c} \bar{Z}\left(\kappa \right)={\left[{\bar{z}}_{1}^{T}\left(\kappa \right),{\bar{z}}_{2}^{T}\left(\kappa \right),\cdots ,{\bar{z}}_{N}^{T}\left(\kappa \right)\right]}^{T} \end{array}$$

$$\begin{array}{c} \bar{H}\left(\kappa \right)={\left[{\bar{H}}_{1}^{T}\left(\kappa \right),{\bar{H}}_{2}^{T}\left(\kappa \right),\cdots ,{\bar{H}}_{N}^{T}\left(\kappa \right)\right]}^{T} \end{array}$$

$$\begin{array}{c} \bar{V}\left(\kappa \right)={\left[{\bar{v}}_{1}^{T}\left(\kappa \right),{\bar{v}}_{2}^{T}\left(\kappa \right),\cdots ,{\bar{v}}_{N}^{T}\left(\kappa \right)\right]}^{T} \end{array}$$

$$\begin{array}{c} \Lambda \left(\kappa \right)=E\left\{\bar{V}\left(\kappa \right){\bar{V}}^{T}\left(\kappa \right)\right\}=diag\left({\bar{R}}_{1}\left(\kappa \right),{\bar{R}}_{2}\left(\kappa \right),\cdots ,{\bar{R}}_{N}\left(\kappa \right)\right) \end{array}$$

Based on the state Equation (1) and the measurement Equation (56), the centralized fusion Kalman filter for the new measurement system is derived as follows:

(57) $$\begin{array}{c} {\hat{\bar{x}}}^{\left(\gamma \right)}\left(\kappa |\kappa \right)=\hat{x}\left(\kappa |\kappa -1\right)+{\bar{K}}^{\left(\gamma \right)}\left(\kappa \right){\tilde{\bar{Z}}}^{\left(\gamma \right)}\left(\kappa |\kappa -1\right) \end{array}$$

(58) $$\begin{array}{c} {\bar{P}}^{\left(\gamma \right)}\left(\kappa |\kappa \right)=P(\kappa |\kappa -1)-{\bar{K}}^{\left(\gamma \right)}\left(\kappa \right){\bar{P}}_{\tilde{\bar{Z}}\tilde{\bar{Z}}}^{\left(\gamma \right)}\left(\kappa \right|\kappa -1){\bar{K}}^{(\gamma )T}\left(\kappa \right) \end{array}$$

where

(59) $$\begin{array}{c} {\bar{K}}^{\left(\gamma \right)}\left(\kappa \right)={\bar{P}}_{\tilde{x}\tilde{\bar{Z}}}^{\left(\gamma \right)}(\kappa |\kappa -1){\bar{P}}_{\tilde{\bar{Z}}\tilde{\bar{Z}}}^{{\left(\gamma \right)}^{-1}}\left(\kappa \right|\kappa -1) \end{array}$$

(60) $$\begin{array}{c} \begin{array}{cc} {\tilde{\bar{Z}}}^{\left(\gamma \right)}\left(\kappa |\kappa -1\right)\; & =\bar{Z}\left(\kappa \right)-\bar{H}\left(\kappa \right)\hat{x}\left(\kappa |\kappa -1\right)\\ & =\bar{H}\left(\kappa \right)\tilde{x}\left(\kappa |\kappa -1\right)+\bar{V}\left(\kappa \right) \end{array} \end{array}$$

(61) $$\begin{array}{c} \begin{array}{cc} {\bar{P}}_{\tilde{x}\tilde{\bar{Z}}}^{\left(\gamma \right)}\left(\kappa |\kappa -1\right) & =E\left\{\tilde{x}\left(\kappa |\kappa -1\right){\tilde{\bar{Z}}}^{(\gamma )T}\left(\kappa |\kappa -1\right)\right\}\\ & =P\left(\kappa |\kappa -1\right)\;{\bar{H}}^{T}\left(\kappa \right) \end{array} \end{array}$$

(62) $$\begin{array}{c} \begin{array}{cc} {\bar{P}}_{\tilde{\bar{Z}}\tilde{\bar{Z}}}^{\left(\gamma \right)}\left(\kappa |\kappa -1\right)\; & =E\left\{{\tilde{\bar{Z}}}^{\left(\gamma \right)}\left(\kappa |\kappa -1\right)\;{\tilde{\bar{Z}}}^{(\gamma )T}\left(\kappa |\kappa -1\right)\right\}\\ & =\bar{H}\left(\kappa \right)\;P\left(\kappa |\kappa -1\right)\;{\bar{H}}^{T}\left(\kappa \right)+\Lambda \left(\kappa \right) \end{array} \end{array}$$

### 5.2. Theorem and Corollaries on Filter Equivalence

Under Assumptions 1 and 2, for system (1)–(2), we have derived the following three filters: the centralized fusion Kalman filter (α), the novel sequential fusion Kalman filter (β), and the centralized fusion Kalman filter (γ). We arrive at the following three conclusions:

**Theorem 1.**  *Filter (β) and filter (γ) are equivalent, i.e., ${\hat{\bar{x}}}^{\left(\gamma \right)}\left(\kappa |\kappa \right)={\hat{\bar{x}}}^{\left(\beta \right)}\left(\kappa |\kappa \right)$ and ${\bar{P}}^{\left(\gamma \right)}\left(\kappa |\kappa \right)={\bar{P}}^{\left(\beta \right)}\left(\kappa |\kappa \right)$. (The proof is provided in Appendix A).*

**Theorem 2.**  *Filter (γ) and filter (α) are equivalent, i.e., ${\hat{\bar{x}}}^{\left(\gamma \right)}\left(\kappa |\kappa \right)={\hat{x}}^{\left(\alpha \right)}\left(\kappa |\kappa \right)$ and ${\bar{P}}^{\left(\gamma \right)}\left(\kappa |\kappa \right)=P^{\left(\alpha \right)}\left(\kappa |\kappa \right)$. (The proof is provided in Appendix B).*

**Corollary 1.**  *From Theorems 1 and 2, it follows that filter (β) and filter (α) are equivalent, i.e., ${\hat{\bar{x}}}^{\left(\beta \right)}\left(\kappa |\kappa \right)={\hat{x}}^{\left(\alpha \right)}\left(\kappa |\kappa \right)$ and ${\bar{P}}^{\left(\beta \right)}\left(\kappa |\kappa \right)=P^{\left(\alpha \right)}\left(\kappa |\kappa \right)$.*

Consequently, it can be concluded that the sequential filter (β), constructed upon the sequential arrival of measurements ${\bar{z}}_{1}\left(\kappa \right),{\bar{z}}_{2}\left(\kappa \right),\cdots ,{\bar{z}}_{N}\left(\kappa \right)$, is equivalent to the centralized fusion filter (α).

## 6. Simulation Research

Experiment 1: Assuming the target moves with constant velocity, the model equations governing its state dynamics are as follows

(63) $$\begin{array}{c} x\left(\kappa \right)=\left[\begin{array}{cc} 1 & T\\ 0 & 1 \end{array}\right]x(\kappa -1)+w(\kappa -1) \end{array}$$

(64) $$\begin{array}{c} z_{i}\left(\kappa \right)=H_{i}\left(\kappa \right)x\left(\kappa \right)+v_{i}\left(\kappa \right),i=1,2,3 \end{array}$$

(65) $$\begin{array}{c} v_{i}\left(\kappa \right)=\theta_{i}w(\kappa -1)+\eta_{i}\left(\kappa \right),i=1,2,3 \end{array}$$

where the sampling time is T=1 s, $x\left(\kappa \right)=\left[\begin{array}{c} x_{1}\\ x_{2} \end{array}\right]$, $x_{1}$ and $x_{2}$ represent the target’s position and velocity, and their units of measurement are m and m/s, respectively. Measurement matrices are denoted as $H_{1}\left(\kappa \right)=\left[\begin{array}{cc} 1 & 0\\ 0 & 1 \end{array}\right]$, $H_{2}\left(\kappa \right)=\left[\begin{array}{cc} 0.8 & 0\\ 0 & 1.1 \end{array}\right]$ and $H_{3}\left(\kappa \right)=\left[\begin{array}{cc} 1.2 & 0\\ 0 & 0.9 \end{array}\right]$, respectively. w(κ−1) and $\eta_{i}\left(\kappa \right)$ denote zero-mean Gaussian white noise sequences with given covariance matrices $\sigma_{w}^{2}$ and $\sigma_{\eta_{i}}^{2}$, respectively, and they are mutually independent.

The process noise w(κ−1) is zero mean Gaussian white noise with covariance matrices $\sigma_{w}^{2}=\left[\begin{array}{cc} T^{3}/3 & T^{2}/2\\ T^{2}/2 & T \end{array}\right]*q$, q=0.5. The measurement noises $v_{i}\left(\kappa \right)$ have a zero mean covariance matrice $R_{i}\left(\kappa \right)=\theta_{i}\sigma_{w}^{2}\theta_{i}^{T}+\sigma_{\eta_{i}}^{2}$. Moreover, $v_{i}\left(\kappa \right)$ and w(κ−1) are correlated, and the strength of the correlation is determined by $\theta_{i}$.

Set $\sigma_{\eta_{1}}^{2}=diag\left(0.25,0.25\right)$, $\sigma_{\eta_{2}}^{2}=diag\left(0.16,0.16\right)$, $\sigma_{\eta_{3}}^{2}=diag\left(0.09,0.09\right)$, $\theta_{1}=diag\left(5,5\right)$, $\theta_{2}=diag\left(4,4\right)$, $\theta_{3}=diag\left(1,1\right)$, the state’s initial value as $x_{0}={\left[2,2\right]}^{T}$, $P_{0}=diag\left(0.1,0.1\right)$.

For each running local Kalman filter, the proposed sequential fusion Kalman filtering algorithm in this paper is applied to obtain the corresponding estimate and its error covariance. The simulation results verify the equivalence between the designed sequential fusion filter and the centralized fusion filter, demonstrating the global optimality of the sequential fusion filter.

This paper adopts the Root Mean Square Error (RMSE) as the performance evaluation metric, and its calculation formula is given as follows:

$$\begin{array}{c} RMSE=\sqrt{\frac{1}{N_{run}}\sum_{i=1}^{N_{run}}{∥\hat{x}\left(\kappa |\kappa \right)-x(\kappa )∥}_{2}^{2}} \end{array}$$

where ${\hat{x}}_{i}\left(\kappa |\kappa \right)$ is the state to be estimated, x(κ) is the system state vector, $N_{run}=100$ is the number of simulation runs, $∥.∥$ denotes the Euclidean norm.

Taking the RMSE of the previous sensor as a reference, the accuracy is computed as follows:

$$\begin{array}{c} \xi \%=\frac{\varepsilon_{i}-\varepsilon_{i-1}}{\varepsilon_{i-1}}\times 100\%,i=2,3\; \end{array}$$

where ε represents the Root Mean Square Error (RMSE) of each sensor.

Table 1 shows that comparing the RMSEs of the three sensors reveals an improvement in state estimation accuracy, verifying the proposed algorithm’s effectiveness.

**Figure 2:**
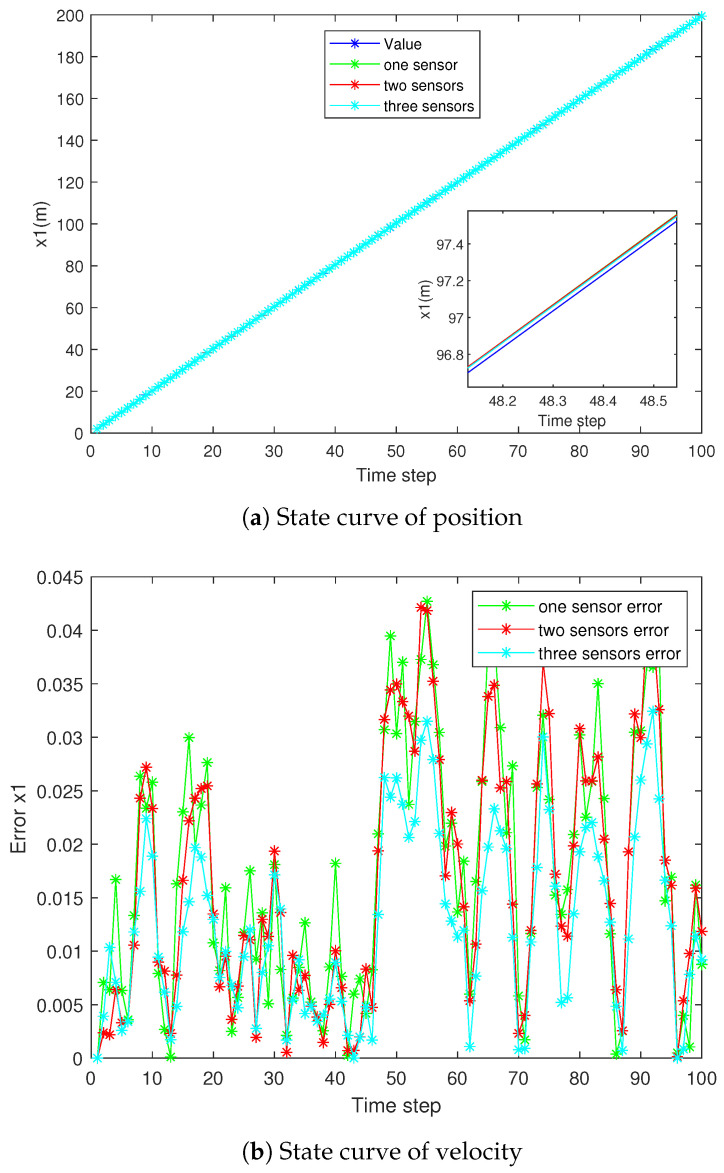
Tracking performance of the three sensors.

**Figure 3:**
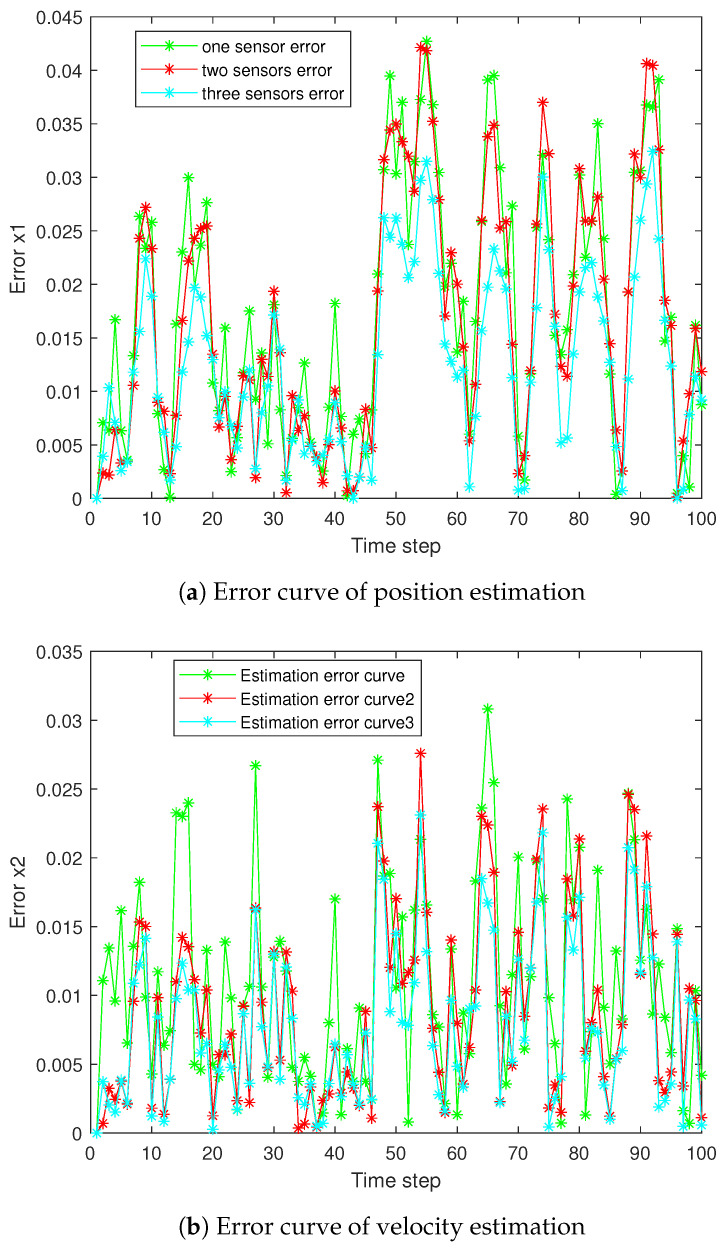
Estimation error of the three sensors.

The state estimation curve and result error curve are shown in Figure 2 and Figure 3, respectively, comparing the estimated values and absolute errors of the three sensors.

**Figure 4:**
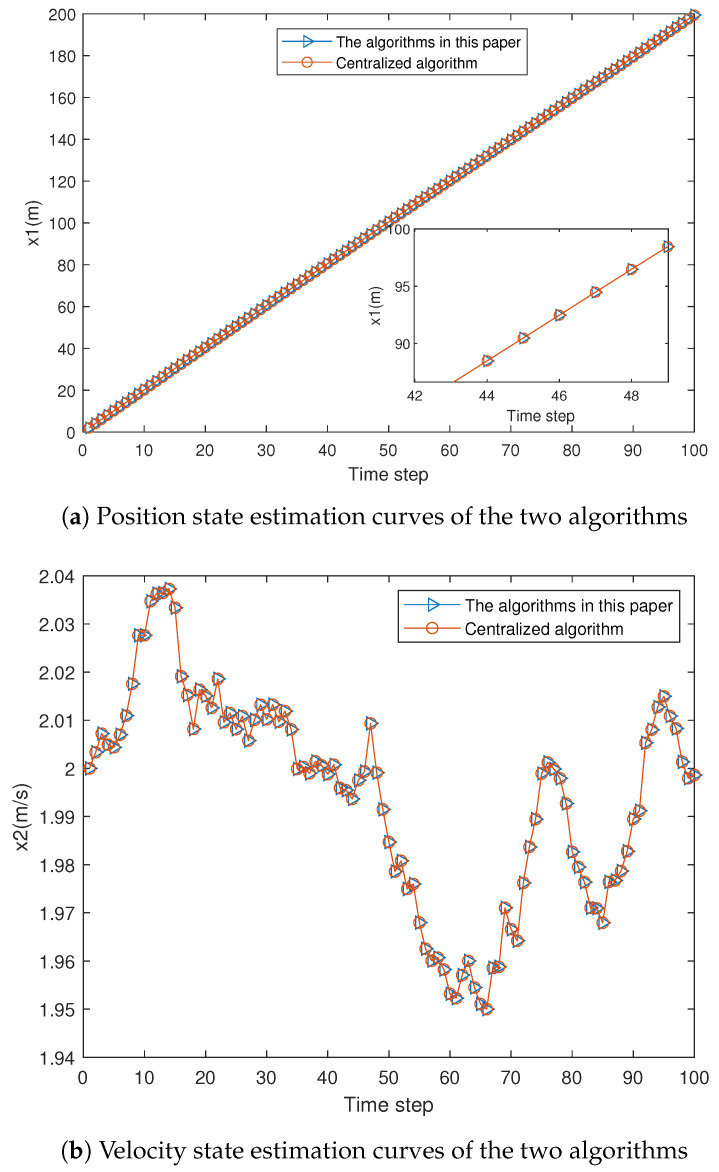
Estimation curves of the two algorithms.

The results in Figure 4 and Table 2 indicate that the proposed algorithm effectively handles multi-sensor systems with noise cross-correlation, achieving the same accuracy as the centralized fusion Kalman filter under correlated noise conditions. This implies that the proposed algorithm is optimal in the linear minimum mean square error sense.

Experiment 2: The state model remains consistent with that of Experiment 1, and the measurement model has been adjusted slightly as shown below:

(66) $$\begin{array}{c} Z(\kappa )=H(\kappa )x(\kappa )+V(\kappa ) \end{array}$$

(67) $$\begin{array}{c} V(\kappa )=\theta w(\kappa -1)+\eta (\kappa ) \end{array}$$

Set measurement matrix $H\left(\kappa \right)=\left[\begin{array}{cc} 1 & 0\\ 0 & 1 \end{array}\right]$, w(κ−1) and η(κ) are mutually independent. The variance matrice of the system process noise w(κ−1) remains as $\sigma_{w}^{2}$ and $\sigma_{\eta }^{2}=diag\left(0.36,0.36\right)$. Moreover, V(κ) are correlated with w(κ−1), the strength of the correlation is determined by θ. The initial value remains $x_{0}={\left[2,2\right]}^{T}$, $P_{0}=diag\left(0.1,0.1\right)$.

By increasing the correlation coefficient θ, we can observe the variation in the trace of the estimation error covariance matrix.

As shown in Table 3, Ignoring correlation means setting the cross-covariance matrix between V(κ) and w(κ−1) to zero. That is, D(κ)=0, and the variation of the filter estimation accuracy is analyzed. Through a horizontal comparison, whether considering noise correlation or ignoring it, the trace of the estimation error covariance matrix gradually decreases with an increasing correlation coefficient, indicating improved estimation accuracy. Through a vertical comparison, regardless of the value of θ, the filter considering noise correlation consistently achieves higher estimation accuracy than the filter that ignores correlation, demonstrating the need to account for noise correlation.

**Remark 5.**  *When the correlation coefficient θ, V(κ) and w(κ−1) are uncorrelated, the designed filters are identical with the same estimation performance, and their covariance matrices are also the same.*

## 7. Conclusions

For the problem of sequential fusion estimation in multi-sensor systems with noise cross-correlation, this paper provides a comprehensive review of the relevant studies conducted by previous researchers. However, existing approaches exhibit certain limitations. To address these issues, a class of globally optimal sequential fusion Kalman filters is proposed. Compared with centralized fusion Kalman filters, the proposed method significantly reduces the computational burden associated with high-dimensional matrix operations and enables real-time estimation, and its global optimality is rigorously proven. The proposed algorithm is applicable not only to multi-sensor systems with noise cross-correlation but also to systems with data delays. Moreover, since noise cross-correlation may also arise in nonlinear multi-sensor systems, extending the proposed algorithm to nonlinear systems with correlated noise will be the focus of future research.

## Figures and Tables

**Table 1:** Estimation error and accuracy improvement for different sensors.

Sensors	RMSE-$x_{1}$ ($10^{-2}$)	RMSE-$x_{2}$ ($10^{-2}$)	Improved-$x_{1}$	Improved-$x_{2}$
Sensors 1	2.08	1.32	×	×
Sensors 2	2.02	1.13	2.9%	14.4%
Sensors 3	1.51	0.97	25.2%	14.2%

“×” indicates that the RMSE of this sensor is displayed for reference only, with no improvement in accuracy.

**Table 2:** Comparison of error covariance matrices estimated by two algorithms.

Algorithm	$P_{x_{1}}\left(\kappa |\kappa \right)$	$P_{x_{2}}\left(\kappa |\kappa \right)$
Centralized fusion	0.0204	0.0058
Sequential fusion	0.0204	0.0058

**Table 3:** Trace of the error covariance matrix for different correlations.

	θ=0	θ=0.2	θ=0.5	θ=0.8	θ=1
Considering correlation	0.2808	0.2488	0.2147	0.1912	0.1796
Ignoring correlation	0.2808	0.3299	0.3051	0.2402	0.1960

## Data Availability

No new data were created or analyzed in this study. Data sharing is not applicable to this article.

## Appendix A

By comparing Equations (53) and (57), to prove the equivalence between filter (γ) and filter (β), it suffices to show that the following equation holds.

(A1) $$\begin{array}{c} {\bar{K}}^{\left(\gamma \right)}\left(\kappa \right){\tilde{\bar{Z}}}^{\left(\gamma \right)}\left(\kappa |\kappa -1\right)=\sum_{l=1}^{N}{\bar{K}}_{l}\left(\kappa \right){\tilde{\bar{z}}}_{l}\left(\kappa |\kappa -1\right) \end{array}$$

(A2) $$\begin{array}{c} {\bar{K}}^{\left(\gamma \right)}\left(\kappa \right){\bar{P}}_{\tilde{\bar{Z}}\tilde{\bar{Z}}}^{\left(\gamma \right)}\left(\kappa \right|\kappa -1){\bar{K}}^{(\gamma )T}\left(\kappa \right)=\sum_{l=1}^{N}{\bar{K}}_{l}\left(\kappa \right){\bar{P}}_{{\tilde{\bar{z}}}_{l}{\tilde{\bar{z}}}_{l}}\left(\kappa |\kappa -1\right){\bar{K}}_{l}^{T}\left(\kappa \right) \end{array}$$

**Proof.** It suffices to prove that Equations (A1) and (A2) hold at time κ for any L∈{1,2,⋯,N}. For the centralized fusion Kalman filter (γ), let

(A3) $$\begin{array}{c} A_{L}\left(\kappa \right)={\bar{H}}_{L}\left(\kappa \right)\tilde{x}\left(\kappa |\kappa -1\right)+{\bar{v}}_{L}\left(\kappa \right),L=1,2,\cdots ,N \end{array}$$

For the sequential fusion Kalman filter (β), an alternative equivalent representation of the innovation ${\tilde{\bar{z}}}_{L}\left(\kappa |\kappa -1\right)$ is given by

(A4) $$\begin{array}{c} \begin{array}{cc} {\tilde{\bar{z}}}_{L}\left(\kappa |\kappa -1\right)\; & ={\bar{H}}_{L}(\kappa ){\tilde{\bar{x}}}_{L-1}\left(\kappa |\kappa \right)+{\bar{v}}_{L}(\kappa )\\ \; & ={\bar{H}}_{L}(\kappa )\left[\tilde{x}\left(\kappa |\kappa -1\right)-\sum_{l=1}^{L-1}{\bar{K}}_{l}\left(\kappa \right){\tilde{\bar{z}}}_{l}\left(\kappa |\kappa -1\right)\right]+{\bar{v}}_{L}(\kappa )\\ \; & =A_{L}(\kappa )-{\bar{H}}_{L}(\kappa )\sum_{l=1}^{L-1}{\bar{K}}_{l}\left(\kappa \right){\tilde{\bar{z}}}_{l}\left(\kappa |\kappa -1\right) \end{array} \end{array}$$

By the Gram–Schmidt orthogonalization process, when L=1, the innovation of the sequential fusion Kalman filter (β) can be equivalently represented by the innovation of the centralized fusion Kalman filter (γ).

(A5) $$\begin{array}{c} \begin{array}{c} {\tilde{\bar{z}}}_{1}\left(\kappa |\kappa -1\right)={\bar{H}}_{1}(\kappa )\tilde{x}\left(\kappa |\kappa -1\right)+{\bar{v}}_{1}(\kappa )\\ =\left[H_{1}\left(\kappa \right)-G_{1,0}\left(\kappa \right){\bar{H}}_{0}\left(\kappa \right)\right]\tilde{x}\left(\kappa |\kappa -1\right)+v_{1}(\kappa )-G_{1,0}(\kappa ){\bar{v}}_{0}(\kappa )\\ =H_{1}(\kappa )\tilde{x}\left(\kappa |\kappa -1\right)+v_{1}(\kappa )\\ ={\tilde{\bar{Z}}}^{\left(\gamma \right)}\left(\kappa |\kappa -1\right) \end{array} \end{array}$$

Therefore, the following equation holds

(A6) $$\begin{array}{c} \begin{array}{c} {\bar{K}}^{\left(\gamma \right)}(\kappa ){\tilde{\bar{Z}}}^{\left(\gamma \right)}\left(\kappa |\kappa -1\right)=E\left\{\tilde{x}\left(\kappa |\kappa -1\right){\tilde{\bar{Z}}}^{(\gamma )T}\left(\kappa |\kappa -1\right)\right\}\\ \;\times {\left[E\left\{{\tilde{\bar{Z}}}^{\left(\gamma \right)}\left(\kappa |\kappa -1\right){\tilde{\bar{Z}}}^{(\gamma )T}\left(\kappa |\kappa -1\right)\right\}\right]}^{-1}{\tilde{\bar{Z}}}^{\left(\gamma \right)}\left(\kappa |\kappa -1\right)\\ \;=E\left\{\tilde{x}\left(\kappa |\kappa -1\right){\tilde{\bar{z}}}_{1}^{T}\left(\kappa |\kappa -1\right)\right\}{\left[E\left\{{\tilde{\bar{z}}}_{1}\left(\kappa |\kappa -1\right){\tilde{\bar{z}}}_{1}^{T}\left(\kappa |\kappa -1\right)\right\}\right]}^{-1}{\tilde{\bar{z}}}_{1}\left(\kappa |\kappa -1\right)\\ \;={\bar{K}}_{1}(\kappa ){\tilde{\bar{z}}}_{1}\left(\kappa |\kappa -1\right) \end{array} \end{array}$$

From Equation (A5), it follows that ${\tilde{\bar{z}}}_{1}\left(k|k-1\right)=A_{1}\left(k\right)$ when L=1. The innovation of the sequential fusion Kalman filter (β) can be equivalently represented by the innovation of the centralized fusion Kalman filter (γ) when L=2.

(A7) $$\begin{array}{cc} {\tilde{\bar{z}}}_{2}\left(\kappa |\kappa -1\right)\; & ={\bar{H}}_{2}\left(\kappa \right)\;{\tilde{\bar{x}}}_{1}\left(\kappa |\kappa -1\right)+{\bar{v}}_{2}\left(\kappa \right)\\ \; & ={\bar{H}}_{2}\left(\kappa \right)\;\left[\tilde{x}\left(\kappa |\kappa -1\right)-{\bar{K}}_{1}\left(\kappa \right)\;{\tilde{\bar{z}}}_{1}\left(\kappa |\kappa -1\right)\right]+{\bar{v}}_{2}\left(\kappa \right)\\ \; & =A_{2}\left(\kappa \right)-{\bar{H}}_{2}\left(\kappa \right)\;{\bar{K}}_{1}\left(\kappa \right)\;{\tilde{\bar{z}}}_{1}\left(\kappa |\kappa -1\right) \end{array}$$

Therefore, it follows that

(A8) $$\begin{array}{c} \left[\begin{array}{c} A_{1}\left(\kappa \right)\\ A_{2}\left(\kappa \right) \end{array}\right]=U\left(\kappa \right)\left[\begin{array}{c} {\tilde{\bar{z}}}_{1}\left(\kappa |\kappa -1\right)\\ {\tilde{\bar{z}}}_{2}\left(\kappa |\kappa -1\right) \end{array}\right] \end{array}$$

where U(k) is reversible, and its expression is as follows

(A9) $$\begin{array}{c} U=\left[\begin{array}{cc} I & 0\\ {\bar{H}}_{2}\left(\kappa \right){\bar{K}}_{1}\left(\kappa \right) & I \end{array}\right] \end{array}$$

Using the equivalence relation of Equation (A8), Equation (A1) of the centralized fusion Kalman filter (γ) equation can be rewritten as follows

(A10) $$\begin{array}{cc} {\bar{K}}^{\left(\gamma \right)}\left(\kappa \right)\;{\tilde{\bar{Z}}}^{\left(\gamma \right)}\left(\kappa |\kappa -1\right)\; & =E\left\{\tilde{x}\left(\kappa |\kappa -1\right){\tilde{\bar{Z}}}^{(\gamma )T}\left(\kappa |\kappa -1\right)\right\}\\ \; & \;\times {\left[E\left\{{\tilde{\bar{Z}}}^{\left(\gamma \right)}\left(\kappa |\kappa -1\right){\tilde{\bar{Z}}}^{(\gamma )T}\left(\kappa |\kappa -1\right)\right\}\right]}^{-1}{\tilde{\bar{Z}}}^{\left(\gamma \right)}\left(\kappa |\kappa -1\right)\\ \; & =E\left\{\tilde{x}\left(\kappa |\kappa -1\right){\left[\begin{array}{c} {\tilde{\bar{z}}}_{1}\left(\kappa |\kappa -1\right)\\ {\tilde{\bar{z}}}_{2}\left(\kappa |\kappa -1\right) \end{array}\right]}^{T}\right\}U^{T}\left(\kappa \right)U^{-T}\left(\kappa \right)\\ \; & \;\times {\left[E\left\{\left[\begin{array}{c} {\tilde{\bar{z}}}_{1}\left(\kappa |\kappa -1\right)\\ {\tilde{\bar{z}}}_{2}\left(\kappa |\kappa -1\right) \end{array}\right]{\left[\begin{array}{c} {\tilde{\bar{z}}}_{1}\left(\kappa |\kappa -1\right)\\ {\tilde{\bar{z}}}_{2}\left(\kappa |\kappa -1\right) \end{array}\right]}^{T}\right\}\right]}^{-1}U^{-1}\left(\kappa \right)U\left(\kappa \right)\left[\begin{array}{c} {\tilde{\bar{z}}}_{1}\left(\kappa |\kappa -1\right)\\ {\tilde{\bar{z}}}_{2}\left(\kappa |\kappa -1\right) \end{array}\right]\\ \; & =E\left\{\tilde{x}\left(\kappa |\kappa -1\right){\left[\begin{array}{c} {\tilde{\bar{z}}}_{1}\left(\kappa |\kappa -1\right)\\ {\tilde{\bar{z}}}_{2}\left(\kappa |\kappa -1\right) \end{array}\right]}^{T}\right\}{\left[E\left\{\left[\begin{array}{c} {\tilde{\bar{z}}}_{1}\left(\kappa |\kappa -1\right)\\ {\tilde{\bar{z}}}_{2}\left(\kappa |\kappa -1\right) \end{array}\right]{\left[\begin{array}{c} {\tilde{\bar{z}}}_{1}\left(\kappa |\kappa -1\right)\\ {\tilde{\bar{z}}}_{2}\left(\kappa |\kappa -1\right) \end{array}\right]}^{T}\right\}\right]}^{-1}\\ \; & \;\times \left[\begin{array}{c} {\tilde{\bar{z}}}_{1}\left(\kappa |\kappa -1\right)\\ {\tilde{\bar{z}}}_{2}\left(\kappa |\kappa -1\right) \end{array}\right] \end{array}$$

**Theorem A1** ([30])**.** *In a multi-sensor linear system, under the conditions that process noise and measurement noise are mutually independent and that measurement noises are mutually independent, the information between successive fusion Kalman filters is independent.* From Equation (54), it follows that $\tilde{x}\left(k|k-1\right)={\tilde{\bar{x}}}_{1}\left(k|k\right)+{\bar{K}}_{1}\left(k\right){\tilde{\bar{z}}}_{1}\left(k|k-1\right)$. Substituting this expression into Equation (A10), and based on Theorem A1, Equation (A10) can be equivalently expressed as

(A11) $$\begin{array}{cc} {\bar{K}}^{\left(\gamma \right)}\left(\kappa \right)\;{\tilde{\bar{Z}}}^{\left(\gamma \right)}\left(\kappa |\kappa -1\right)\; & =E\left\{\tilde{x}\left(\kappa |\kappa -1\right)\left[{\tilde{\bar{z}}}_{1}^{T}\left(\kappa |\kappa -1\right),{\tilde{\bar{z}}}_{2}^{T}\left(\kappa |\kappa -1\right)\right]\right\}\\ \; & \times {\left[\begin{array}{cc} P_{{\bar{z}}_{1}{\bar{z}}_{1}}^{-1}\left(\kappa |\kappa -1\right) & 0\\ 0 & P_{{\bar{z}}_{2}{\bar{z}}_{2}}^{-1}\left(\kappa |\kappa -1\right) \end{array}\right]}^{-1}\left[\begin{array}{c} {\tilde{\bar{z}}}_{1}\left(\kappa |\kappa -1\right)\\ {\tilde{\bar{z}}}_{2}\left(\kappa |\kappa -1\right) \end{array}\right]\\ \; & =\left[{\bar{P}}_{{\tilde{x}}_{1}{\tilde{\bar{z}}}_{1}}\left(\kappa |\kappa -1\right){\bar{P}}_{{\tilde{\bar{z}}}_{1}{\tilde{\bar{z}}}_{1}}^{-1}\left(\kappa |\kappa -1\right),\;{\bar{P}}_{{\tilde{\bar{x}}}_{1}{\tilde{\bar{z}}}_{2}}\left(\kappa |\kappa -1\right){\bar{P}}_{{\tilde{\bar{z}}}_{2}{\tilde{\bar{z}}}_{2}}^{-1}\left(\kappa |\kappa -1\right)\right]\left[\begin{array}{c} {\tilde{\bar{z}}}_{1}\left(\kappa |\kappa -1\right)\\ {\tilde{\bar{z}}}_{2}\left(\kappa |\kappa -1\right) \end{array}\right]\\ \; & ={\bar{K}}_{1}\left(\kappa \right)\;{\tilde{\bar{z}}}_{1}\left(\kappa |\kappa -1\right)+{\bar{K}}_{2}\left(\kappa \right)\;{\tilde{\bar{z}}}_{2}\left(\kappa |\kappa -1\right) \end{array}$$

Assumption Equation (A1) holds for L=N−1. One will prove that (A1) also holds for L=N; in another words, ${\hat{\bar{x}}}^{\left(\beta \right)}\left(\kappa |\kappa \right)={\hat{\bar{x}}}^{\left(\gamma \right)}\left(\kappa |\kappa \right)$ and ${\bar{P}}^{\left(\beta \right)}\left(\kappa |\kappa \right)={\bar{P}}^{\left(\gamma \right)}\left(\kappa |\kappa \right)$. Set ${\bar{A}}_{1}\left(\kappa \right)={\left[A_{1}^{T}\left(\kappa \right),A_{2}^{T}\left(\kappa \right),\cdots ,A_{N-1}^{T}\left(\kappa \right)\right]}^{T}$, ${\bar{A}}_{2}\left(\kappa \right)=A_{N}\left(\kappa \right)$, ${\bar{C}}_{1}\left(\kappa \right)=[{\tilde{\bar{z}}}_{1}^{T}(\kappa |\kappa -1),{\tilde{\bar{z}}}_{2}^{T}(\kappa |\kappa -1),\cdots$, ${\tilde{\bar{z}}}_{N-1}^{T}{\left(\kappa |\kappa -1\right)]}^{T}$, ${\bar{C}}_{2}\left(\kappa \right)={\tilde{\bar{z}}}_{N}^{T}\left(\kappa |\kappa -1\right)$. According to Equation (A4), the equivalence relations between ${\bar{A}}_{1}\left(\kappa \right)$ and ${\bar{C}}_{1}\left(\kappa \right)$ are as follows

(A12) $$\begin{array}{c} {\bar{A}}_{1}\left(\kappa \right)={\bar{U}}_{1}\left(\kappa \right){\bar{C}}_{1}\left(\kappa \right) \end{array}$$

where ${\bar{U}}_{1}\left(\kappa \right)$ is reversible, and its expression is as follows

(A13) $$\begin{array}{c} {\bar{U}}_{1}\left(\kappa \right)=\left[\begin{array}{ccccc} I & 0 & \cdots & 0 & 0\\ {\bar{H}}_{2}\left(\kappa \right){\bar{K}}_{1}\left(\kappa \right) & I & \cdots & \vdots & 0\\ {\bar{H}}_{3}\left(\kappa \right){\bar{K}}_{1}\left(\kappa \right) & {\bar{H}}_{3}\left(\kappa \right){\bar{K}}_{2}\left(\kappa \right) & \ddots & 0 & \vdots \\ \vdots & \vdots & \vdots & I & 0\\ {\bar{H}}_{N-1}\left(\kappa \right){\bar{K}}_{1}\left(\kappa \right) & {\bar{H}}_{N-1}\left(\kappa \right){\bar{K}}_{2}\left(\kappa \right) & \cdots & {\bar{H}}_{N-1}\left(\kappa \right){\bar{K}}_{N-2}\left(\kappa \right) & I \end{array}\right] \end{array}$$

Similar to Equation (A8), the following equation is obtained

(A14) $$\begin{array}{c} \left[\begin{array}{c} {\bar{A}}_{1}\left(\kappa \right)\\ {\bar{A}}_{2}\left(\kappa \right) \end{array}\right]=\bar{U}\left(\kappa \right)\left[\begin{array}{c} {\bar{C}}_{1}\left(\kappa \right)\\ {\bar{C}}_{2}\left(\kappa \right) \end{array}\right] \end{array}$$

where

$$\begin{array}{c} \bar{U}\left(\kappa \right)=\left[\begin{array}{cc} {\bar{U}}_{1}\left(\kappa \right) & \Omega (\kappa )\\ \Delta (\kappa ) & I \end{array}\right] \end{array}$$

$$\begin{array}{c} \Delta \left(\kappa \right)=[{\bar{H}}_{N}\left(\kappa \right){\bar{K}}_{1}\left(\kappa \right),{\bar{H}}_{N}\left(\kappa \right){\bar{K}}_{2}\left(\kappa \right),\cdots {\bar{H}}_{N}\left(\kappa \right){\bar{K}}_{N-1}\left(\kappa \right)] \end{array}$$

$$\begin{array}{c} \Omega \left(\kappa \right)={\left[0,0,\cdots ,0\right]}^{T} \end{array}$$

Then, similar to the case of L=2, the same derivation process as (A10) and (A11) can be implemented. One can draw the conclusion that the estimation accuracy of the sequential and centralized fusion filters is same for L=N. From the above proof, it follows that ${\hat{\bar{x}}}^{\left(\gamma \right)}\left(\kappa |\kappa \right)={\hat{\bar{x}}}^{\left(\beta \right)}\left(\kappa |\kappa \right)$. Similarly, by applying the same reasoning, one draws the conclusion that ${\bar{P}}^{\left(\gamma \right)}\left(\kappa |\kappa \right)={\bar{P}}^{\left(\beta \right)}\left(\kappa |\kappa \right)$ can be proven. The proof is completed.  □

## Appendix B

**Proof.** According to Equation (37) and the definition of $\bar{H}\left(\kappa \right)$, H(κ) can be expressed as

(A15) $$\begin{array}{c} H\left(\kappa \right)=\left[\begin{array}{c} H_{1}\left(\kappa \right)\\ H_{2}\left(\kappa \right)\\ \vdots \\ H_{N}\left(\kappa \right) \end{array}\right]=M\left(\kappa \right)\left[\begin{array}{c} {\bar{H}}_{1}\left(\kappa \right)\\ {\bar{H}}_{2}\left(\kappa \right)\\ \vdots \\ {\bar{H}}_{N}\left(\kappa \right) \end{array}\right]+N\left(\kappa \right){\bar{H}}_{0}\left(\kappa \right) \end{array}$$

where

(A16) $$\begin{array}{c} M\left(\kappa \right)=\left[\begin{array}{ccccc} I & 0 & 0 & \cdots & 0\\ G_{2,1}\left(\kappa \right) & I & 0 & \cdots & 0\\ G_{3,1}\left(\kappa \right) & G_{3,2}\left(\kappa \right) & \ddots & \vdots & \vdots \\ \vdots & \vdots & \vdots & I & 0\\ G_{N,1}\left(\kappa \right) & G_{N,2}\left(\kappa \right) & \cdots & G_{N,N-1}\left(\kappa \right) & I \end{array}\right] \end{array}$$

(A17) $$\begin{array}{cc} N(\kappa ) & =\left[\begin{array}{c} G_{1,0}\left(\kappa \right)\\ G_{2,0}\left(\kappa \right)\\ \vdots \\ G_{N,0}\left(\kappa \right) \end{array}\right]=-\left[\begin{array}{c} B_{1}^{T}\left(\kappa \right)\\ B_{2}^{T}\left(\kappa \right)\\ \vdots \\ B_{N}^{T}\left(\kappa \right) \end{array}\right]P^{-1}\left(\kappa |\kappa -1\right)\\ \; & =-D^{T}\left(\kappa \right)P^{-1}\left(\kappa |\kappa -1\right) \end{array}$$

Then, $\bar{H}\left(\kappa \right)$ can be expressed as

(A18) $$\begin{array}{c} \bar{H}\left(\kappa \right)=M^{-1}\left(\kappa \right)\left[H\left(\kappa \right)-N\left(\kappa \right){\bar{H}}_{0}\left(\kappa \right)\right] \end{array}$$

Similarly, $\bar{V}\left(\kappa \right)$ can be expressed as

(A19) $$\begin{array}{c} \bar{V}\left(\kappa \right)=M^{-1}\left(\kappa \right)\left[V\left(\kappa \right)-N\left(\kappa \right){\bar{v}}_{0}\left(\kappa \right)\right] \end{array}$$

Then, V(κ) can be expressed as

(A20) $$\begin{array}{c} V\left(\kappa \right)=M\left(\kappa \right)\bar{V}\left(\kappa \right)+N\left(\kappa \right){\bar{v}}_{0}\left(\kappa \right) \end{array}$$

Due to the Gram–Schmidt orthogonalization process, $\bar{V}\left(\kappa \right)$ and ${\bar{v}}_{0}\left(\kappa \right)$ are no longer correlated, and it follows that

(A21) $$\begin{array}{c} R\left(\kappa \right)=M\left(\kappa \right)\Lambda \left(\kappa \right)M^{T}\left(\kappa \right)+D^{T}\left(\kappa \right){\bar{R}}_{0}^{-1}\left(\kappa \right)D\left(\kappa \right) \end{array}$$

and

(A22) $$\begin{array}{c} \Lambda \left(\kappa \right)=M^{-1}\left(\kappa \right)\left[R\left(\kappa \right)-D^{T}\left(\kappa \right){\bar{R}}_{0}^{-1}\left(\kappa \right)D\left(\kappa \right)\right]M^{(-1)T}\left(\kappa \right) \end{array}$$

To prove the equivalence between ${\hat{x}}^{\left(\alpha \right)}\left(\kappa |\kappa \right)={\hat{\bar{x}}}^{\left(\gamma \right)}\left(\kappa |\kappa \right)$ and $P^{\left(\alpha \right)}\left(\kappa |\kappa \right)={\bar{P}}^{\left(\gamma \right)}\left(\kappa |\kappa \right)$, it suffices to show that the following equation holds.

(A23) $$\begin{array}{c} {\bar{K}}^{\left(\gamma \right)}\left(\kappa \right){\tilde{\bar{Z}}}^{\left(\gamma \right)}\left(\kappa |\kappa -1\right)=K^{\left(\alpha \right)}\left(\kappa \right){\tilde{Z}}^{\left(\alpha \right)}\left(\kappa |\kappa -1\right) \end{array}$$

(A24) $$\begin{array}{c} {\bar{K}}^{\left(\gamma \right)}\left(\kappa \right){\bar{P}}_{\tilde{\bar{Z}}\tilde{\bar{Z}}}^{\left(\gamma \right)}(\kappa |\kappa -1){\bar{K}}^{(\gamma )T}\left(\kappa \right)=K^{\left(\alpha \right)}\left(\kappa \right)P_{\tilde{Z}\tilde{Z}}^{\left(\alpha \right)}\left(\kappa \right|\kappa -1)K^{(\alpha )T}\left(\kappa \right) \end{array}$$

Combining Equations (61) and (A18), the covariance matrix ${\bar{P}}_{\tilde{x}\tilde{\bar{Z}}}^{\left(\gamma \right)}\left(\kappa \right|\kappa -1)$ can be equivalently expressed as

(A25) $$\begin{array}{cc} {\bar{P}}_{\tilde{x}\tilde{\bar{Z}}}^{\left(\gamma \right)}\left(\kappa |\kappa -1\right)\; & =P\left(\kappa |\kappa -1\right){\bar{H}}^{T}\left(\kappa \right)\\ \; & =\left[P\left(\kappa |\kappa -1\right)H^{T}\left(\kappa \right)+D\left(\kappa \right)\right]M^{(-1)T}\left(\kappa \right)\\ \; & =P_{\tilde{x}\tilde{Z}}\left(\kappa |\kappa -1\right)M^{(-1)T}\left(\kappa \right) \end{array}$$

Combining Equations (62), (A18) and (A22), the covariance matrix ${\bar{P}}_{\tilde{\bar{Z}}\tilde{\bar{Z}}}^{\left(\gamma \right)}\left(\kappa \right|\kappa -1)$ can be equivalently expressed as

(A26) $$\begin{array}{c} \begin{array}{c} {\bar{P}}_{\tilde{\bar{Z}}\tilde{\bar{Z}}}^{\left(\gamma \right)}(\kappa |\kappa -1)=\bar{H}(\kappa )P(\kappa |\kappa -1){\bar{H}}^{T}(\kappa )+\Lambda \left(\kappa \right)\\ \;\;=M^{-1}\left(\kappa \right)[H\left(\kappa \right)P\left(\kappa |\kappa -1\right)H^{T}(\kappa )+H(\kappa )D(\kappa )\\ \;\;+D^{T}\left(k\right)H^{T}\left(k\right)+R\left(\kappa \right)]M^{(-1)T}(\kappa )\\ \;\;=M^{-1}(\kappa )P_{\tilde{Z}\tilde{Z}}\left(\kappa |\kappa -1\right)M^{(-1)T}(\kappa ) \end{array} \end{array}$$

Therefore, the result is

(A27) $$\begin{array}{cc} {\bar{K}}^{\left(\gamma \right)}\left(\kappa \right)\; & ={\bar{P}}_{\tilde{x}\tilde{\bar{Z}}}^{\left(\gamma \right)}\left(\kappa |\kappa -1\right)\;{\bar{P}}_{\tilde{\bar{Z}}\tilde{\bar{Z}}}^{{\left(\gamma \right)}^{-1}}\left(\kappa |\kappa -1\right)\\ \; & =K^{\left(\alpha \right)}\left(\kappa \right)\;M\left(\kappa \right) \end{array}$$

Similarly, by combining Equations (60), (A18), and (A19), the innovation ${\tilde{\bar{Z}}}^{\left(\gamma \right)}\left(\kappa |\kappa -1\right)$ can be equivalently expressed as

(A28) $$\begin{array}{cc} {\tilde{\bar{Z}}}^{\left(\gamma \right)}\left(\kappa |\kappa -1\right) & =\bar{H}\left(\kappa \right)\tilde{x}\left(\kappa |\kappa -1\right)+\bar{V}\left(\kappa \right)\\ \; & =M^{-1}\left(\kappa \right)\left[H\left(\kappa \right)-N\left(\kappa \right){\bar{H}}_{0}\left(\kappa \right)\right]\tilde{x}\left(\kappa |\kappa -1\right)\\ \; & +M^{-1}\left(\kappa \right)\left[V\left(\kappa \right)-N\left(\kappa \right){\bar{v}}_{0}\left(\kappa \right)\right]\\ \; & =M^{-1}\left(\kappa \right){\tilde{Z}}^{\left(\alpha \right)}\left(\kappa |\kappa -1\right) \end{array}$$

From Equations (A27) and (A28), the validity of Equation (A23) can be established, and one draws the conclusion that ${\hat{x}}^{\left(\alpha \right)}\left(\kappa |\kappa \right)={\hat{\bar{x}}}^{\left(\gamma \right)}\left(\kappa |\kappa \right)$ and ${\bar{P}}^{\left(\gamma \right)}\left(\kappa |\kappa \right)=P^{\left(\alpha \right)}\left(\kappa |\kappa \right)$. The proof of Theorem 2 is complete.  □
